# Using multi-scale genomics to associate poorly annotated genes with rare diseases

**DOI:** 10.1186/s13073-023-01276-2

**Published:** 2024-01-04

**Authors:** Christina Canavati, Dana Sherill-Rofe, Lara Kamal, Idit Bloch, Fouad Zahdeh, Elad Sharon, Batel Terespolsky, Islam Abu Allan, Grace Rabie, Mariana Kawas, Hanin Kassem, Karen B. Avraham, Paul Renbaum, Ephrat Levy-Lahad, Moien Kanaan, Yuval Tabach

**Affiliations:** 1 Department of Developmental Biology and Cancer Research, Institute of Medical Research - Israel-Canada, The Hebrew University of Jerusalem, Jerusalem, 9112102 Israel; 2 Molecular Genetics Lab, Istishari Arab Hospital, Ramallah, Palestine; 3 Department of Human Molecular Genetics and Biochemistry, Faculty of Medicine and Sagol School of Neuroscience, Tel Aviv University, Tel Aviv, 6997801 Israel; 4 Medical Genetics Institute, Shaare Zedek Medical Center, Jerusalem, 91031 Israel; 5 Faculty of Medicine, The Hebrew University of Jerusalem, Jerusalem, 9112102 Israel; 6 Hereditary Research Laboratory and Department of Life Sciences, Bethlehem University, Bethlehem, 72372 Palestine

**Keywords:** EvORanker, Gene-based prioritization, *DLGAP2*, *LPCAT3*

## Abstract

**Background:**

Next-generation sequencing (NGS) has significantly transformed the landscape of identifying disease-causing genes associated with genetic disorders. However, a substantial portion of sequenced patients remains undiagnosed. This may be attributed not only to the challenges posed by harder-to-detect variants, such as non-coding and structural variations but also to the existence of variants in genes not previously associated with the patient’s clinical phenotype. This study introduces EvORanker, an algorithm that integrates unbiased data from 1,028 eukaryotic genomes to link mutated genes to clinical phenotypes.

**Methods:**

EvORanker utilizes clinical data, multi-scale phylogenetic profiling, and other omics data to prioritize disease-associated genes. It was evaluated on solved exomes and simulated genomes, compared with existing methods, and applied to 6260 knockout genes with mouse phenotypes lacking human associations. Additionally, EvORanker was made accessible as a user-friendly web tool.

**Results:**

In the analyzed exomic cohort, EvORanker accurately identified the “true” disease gene as the top candidate in 69% of cases and within the top 5 candidates in 95% of cases, consistent with results from the simulated dataset. Notably, EvORanker outperformed existing methods, particularly for poorly annotated genes. In the case of the 6260 knockout genes with mouse phenotypes, EvORanker linked 41% of these genes to observed human disease phenotypes. Furthermore, in two unsolved cases, EvORanker successfully identified *DLGAP2* and *LPCAT3* as disease candidates for previously uncharacterized genetic syndromes.

**Conclusions:**

We highlight clade-based phylogenetic profiling as a powerful systematic approach for prioritizing potential disease genes. Our study showcases the efficacy of EvORanker in associating poorly annotated genes to disease phenotypes observed in patients. The EvORanker server is freely available at https://ccanavati.shinyapps.io/EvORanker/.

**Supplementary Information:**

The online version contains supplementary material available at 10.1186/s13073-023-01276-2.

## Background

The study of Mendelian disorders remains a gold standard for understanding gene function and linking a gene to a particular phenotype [1]. Next-generation sequencing technologies (NGS) have revolutionized disease-gene discovery; however, most human genes are yet to be associated with a specific phenotype: Out of ~20,000 human genes, only 4900 genes have an associated phenotype in Online Mendelian Inheritance in Man (OMIM) (as of January 22, 2023) [2]. Analyses of evolutionary constraints of human genes and models from mouse genetics suggest that the genetic basis of at least 10,000 Mendelian disorders awaits discovery [1].

A typical human whole-exome sequencing (WES) or whole-genome sequencing (WGS) study yields thousands of single nucleotide variants, indels, and copy number variants [3]. After filtering out frequent variants, a handful of in silico methods are used to estimate the pathogenicity of the variants. These estimates are based on evolutionary conservation, genomic position, structural features, and predicted function (e.g., impact on regulatory, splicing, or protein level) [4–7]. However, prioritizing variants solely based on predicted pathogenicity and rarity may not lead to identifying the underlying disease-causing gene. Hence, numerous gene-based computational methods have been developed to prioritize candidate genes contributing to the patient’s disease phenotypes. These methods incorporate patients’ phenotypic information and omics datasets such as protein-protein interaction, co-expression analysis, cross-species phenotypic similarity analysis, and primary literature in order to provide users with further hints about genes that merit further investigation [8–12]. Recent approaches incorporate artificial intelligence for candidate gene diagnosis [13, 14]. Nevertheless, the majority of these approaches depend on existing data and as such work well on studied genes (i.e., the rich get richer phenomenon). However, for poorly annotated genes, their performance may decline. Within the scope of this research, poorly annotated genes are defined as those lacking associations with specific phenotypes in the OMIM database [2]. This absence of phenotype correlations underscores a scarcity of available data pertaining to their characteristics, ontology, and interactions [1].

One unbiased approach that can infer novel gene function is phylogenetic profiling (PP) [15]. PP identifies functionally related genes and protein-protein interactions using comparative genomics [16, 17]. The phylogenetic profile of a gene describes the pattern of conservation of its orthologs in a set of genomes [15]. Those patterns of conservation of protein sequences along evolution reflect protein function [18, 19], interaction with other proteins [17], and the crosstalk between the organism and the environment [20]. PP relies on the well-established hypothesis that if two or more genes share a similar phylogenetic profile, then they may be functionally related [15, 21–23]. This can further be implemented to annotate uncharacterized genes to a putative function based on the similarity of their PP with those of well-annotated genes [24]. Previous studies using PP have succeeded in identifying new functional gene associations, novel disease-causing genes, and new pathway components [21, 22, 25, 26].

The phylogenetic profile of a gene was originally described as a binary representation of the presence or absence of its orthologs across eukaryotic species [15, 27, 28]. However, applying a binary representation to eukaryotes might not fully address the intricacy of eukaryotic protein evolution. Across the tree of life, the sequence similarity between two orthologs is a complex function of their evolutionary distance and the variable selective pressures forced upon them. Therefore, several methods such as normalized phylogenetic profiling (NPP) have been developed [21, 22, 24, 29]. NPP uses a continuous metric of conservation, offering an alternative to the binary scoring system. NPP has successfully revealed novel genes in various pathways and human genetic diseases including cancer [21, 25, 26, 29]. Recently, we found that functionally-related genes can show a strong signal of correlated evolution within specific clades (e.g., animals, mammals, plants, fungi) or segments of the tree of life. These local co-evolution signals better reflect the complexity of pathways and protein evolution. Analyzing correlated evolution among genes both across and in part of the tree of life hence improves our ability to reveal the function of genes in different pathways [24, 29, 30]. This “clade-wise” NPP approach was used to identify novel DNA repair genes [29, 30] and to map potential drugs for *MECP2* [31] and *ACE2*-associated disorders [32].

**Fig. 1:**
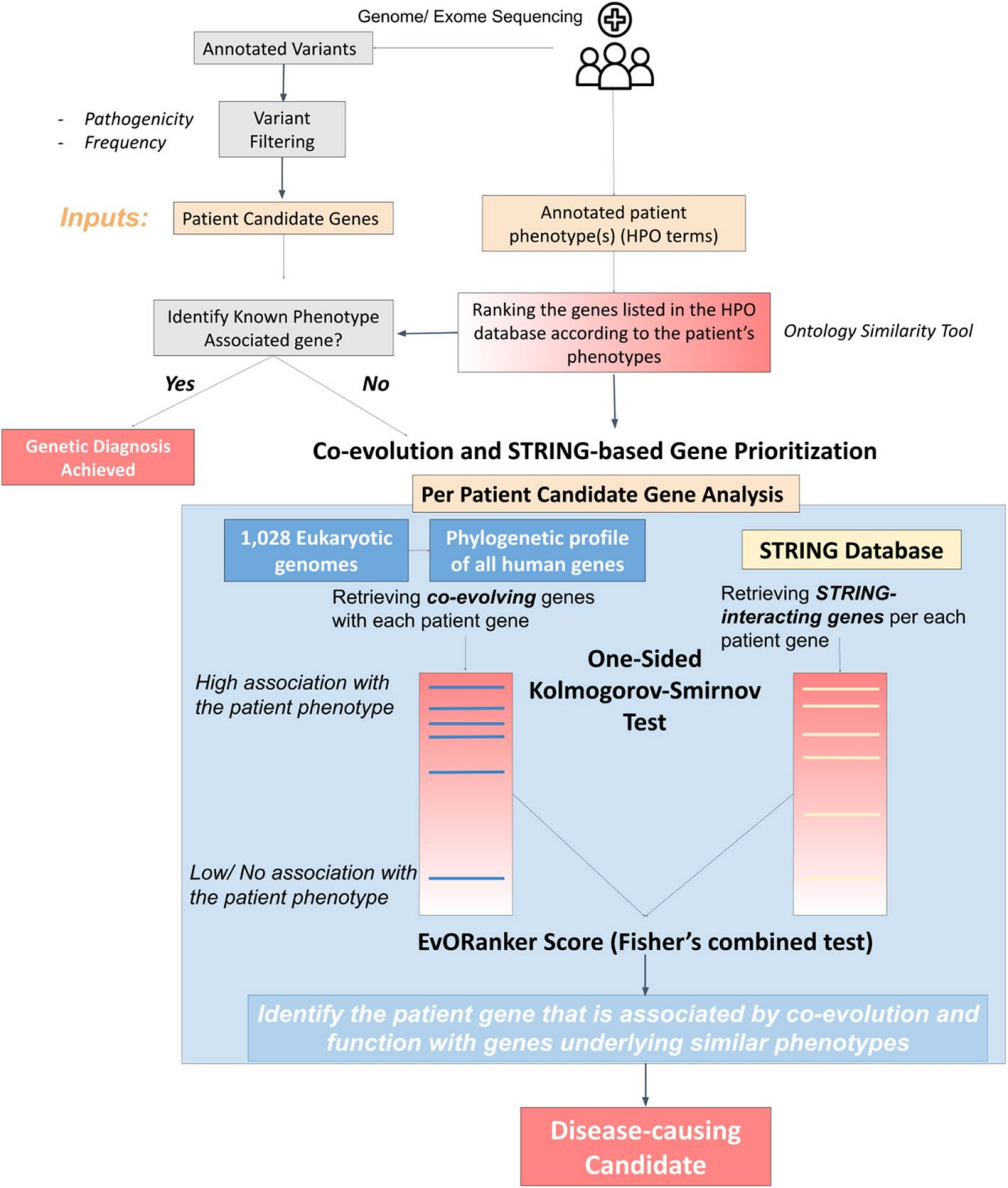
Graphical abstract of the EvORanker pipeline. Starting from a list of annotated variants obtained from a patient’s exome/genome sequencing data and following variant filtering, a list of predicted *patient candidate genes* harboring putatively pathogenic variants are input to EvORanker. The second input is the HPO terms corresponding to the patient’s phenotypes. The first step of the pipeline is to rank the genes listed in the HPO database according to the input HPO terms using the OntologySimilarity tool. If any of the patient candidate genes is a known disease-causing gene or ranked high using *OntologySimilarity*, then a genetic diagnosis is achieved. If not, then each patient candidate gene in addition to the ranked HPO gene list is input into a co-evolution and STRING-based algorithm. The algorithm analyzes two lists of genes, the co-evolving and STRING-interacting genes with each patient candidate gene. A one-sided Kolmogorov-Smirnov (K-S) test is then used to test if the co-evolving and interacting genes rank significantly high within the patient’s *phenotype-related genes*. The *p*-values obtained from running the K-S test using each dataset are combined using Fisher’s combined test. The output is a list of patient candidate genes ranked based on Fisher’s combined test *p*-values (from more significant to less significant). A disease-causing candidate is identified among the patient genes where a significant number of co-evolving and/or interacting genes are enriched towards the genes highly related to the patient’s input phenotypes relative to the genes that are unrelated

In this study, we introduce a gene-prioritization tool, EvORanker (Ev: Evolution, O: Omics) (Fig. 1). EvORanker integrates clade-wise NPP with omics data (e.g., protein-protein interaction, and co-expression data) obtained from the STRING database [33] to associate a gene variant present in a patient with the patient’s phenotypes. Our primary objective is to establish an unbiased framework to resolve unsolved exomes/genomes by linking genes and especially poorly annotated genes to the disease phenotype observed in patients. Furthermore, we aimed to assess EvORanker’s efficacy in identifying candidate disease genes that lack human annotation.

Mouse knockout data serves as a valuable resource for comprehending gene function [34]. We, therefore, concentrated on genes with knockout phenotypes in mice that lack corresponding human annotation and demonstrate EvOranker’s ability to identify disease candidates with limited information. The results of our analysis showed that EvORanker was able to identify disease genes that were ranked low by other gene-based methods, demonstrating the complementarity of EvORanker to those of other gene-based tools. Moreover, we employed EvORanker to investigate two patients with unresolved genetic syndromes. Our analysis revealed *DLGAP2* and *LPCAT3* as potential candidates for disease-causing genes. Notably, only clade-based NPP analysis was able to detect *LPCAT3* as a disease candidate. To enhance its practical utility, we designed EvORanker as a user-friendly gene-prioritization web tool that can be used by researchers and clinicians studying genetic disorders.

## Methods

### The patient exome database

**Fig. 2:**
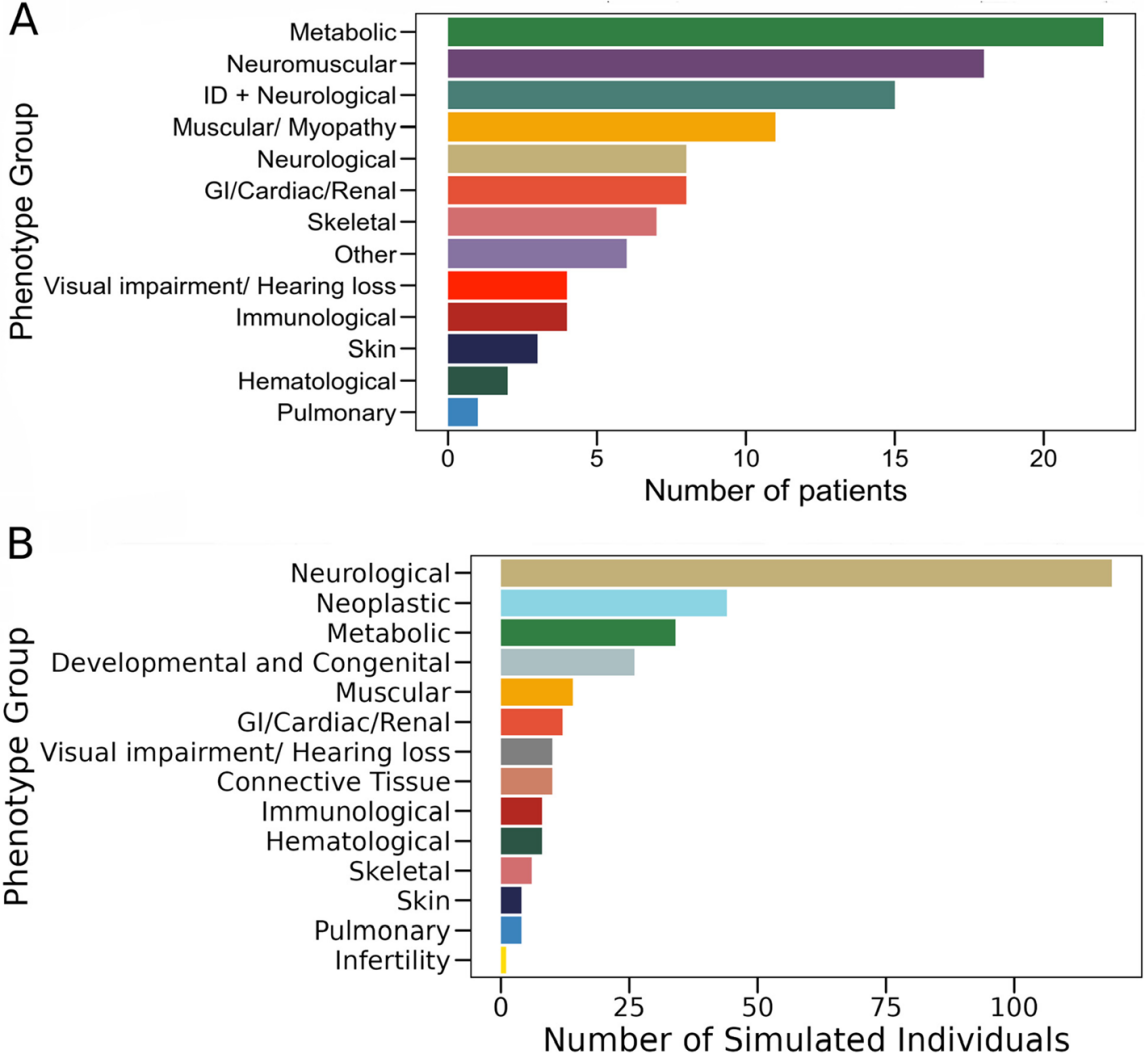
Phenotypic diversity in **A** a cohort of 109 patients from the exome database and **B** a simulated dataset of 300 individuals with 300 pathogenic variants from ClinVar inserted into their genomes. The patients exhibit a wide range of phenotypes. Notably, various shared phenotypes, especially related to metabolic and neurological diseases, are observed among the patients. Key: ID, intellectual disability; GI, gastrointestinal disorders

For the purpose of benchmarking EvORanker, we gathered sequencing data of patients who sought clinical whole-exome sequencing at Istishari Arab Hospital in Ramallah for diagnostic purposes (Additional file 1: Table S1). Each participant presented with a distinct clinical anomaly and was referred to Istishari Hospital by their respective physicians from various Palestinian regions to undergo exome sequencing for the identification of genetic causes. We established a database of 109 patient exomes who had received a known molecular diagnosis, some of which have been published [35–37]. All of the variants identified to be disease-causing in those patients had been reported as pathogenic/likely pathogenic in the ClinVar database [38]. The patients in this cohort exhibit diverse phenotypes (e.g., skeletal, immunological, neurological, and metabolic) (Fig. 2A). The majority of the patients were born to consanguineous parents. Each patient had been clinically diagnosed with a different rare Mendelian disease (Additional file 1: Table S1), of which 91 followed an autosomal or X-linked recessive and 18 followed an autosomal or X-linked dominant mode of inheritance. Familial segregation analysis had been performed for each of the patients’ families further confirming the diagnosis. In addition to the 109 exomes, we applied EvORanker on two exomes of patients (II-3, Fig. 10A, II-4, Fig. 11A) that had not received a molecular diagnosis. Written informed consent was obtained from all participants, or their parents, before their inclusion in the study. All the study participants, or their parents, provided permission to access their medical records.

### Simulation of cases with disease-causing gene variants

We assessed our approach and compared it to other methods by simulating scenarios where mutations in disease-causing genes exist within a genome. VCF files of 300 individual genomes were downloaded from the 1000 Genome Project [39], providing a diverse representation of human populations (http://ftp.1000genomes.ebi.ac.uk/vol1/ftp/release/20100804/) [40]. Variant annotation was performed using ANNOVAR [41], and our variant filtering pipeline was applied (see Table 1).

**Table 1:** Routine variant filtering criteria

	**Autosomal and X-linked recessive mode**	**Autosomal and X-linked dominant mode**
	**Filtering criteria**	**Filtering criteria**
**1. Variant frequency**		
gnomAD [42]	<= 0.02	< 0.001
AF_popmax [42]	<= 0.02	< 0.001
In-house exome database	<= 0.02	< 0.001
**2. Splicing and synonymous variants**		
dbscSNV_RF_SCORE and dbscSNV_ADA_SCORE [43]	dbscSNV_RF_SCORE >= 0.5 or dbscSNV_ADA_SCORE >= 0.5 or SpliceAI >= 0.5	dbscSNV_RF_SCORE >= 0.5 or dbscSNV_ADA_SCORE >= 0.5 or SpliceAI >= 0.5
SpliceAI [44]		
**3. Nonsynonymous variants**		
Polyphen2_HDIV_score [5]	Polyphen2_HDIV_score >= 0.5 or REVEL score >= 0.5 or SIFT score <= 0.5	Polyphen2_HDIV_score >= 0.5 or REVEL score >= 0.5 or SIFT score <= 0.5
REVEL score [6]		
SIFT score [45]		

For each of the 300 genomes, we introduced exonic or splicing pathogenic/likely pathogenic variants from the ClinVar database [38], excluding disease genes already found in the 109-real exome patient dataset. These 300 ClinVar variants were randomly selected from genes associated with diverse disease phenotypes, including complex neoplastic disorders (Fig. 2B). Among these variants, 181 followed an autosomal or X-linked recessive mode of inheritance, while 119 variants followed a dominant inheritance pattern. In instances where the mode of inheritance for a ClinVar pathogenic variant was uncertain, or if it could be attributed to both inheritance modes, or if it was not reported in affected patients, we randomly assigned the mode of inheritance. Subsequently, we randomly integrated these 300 sampled variants into the 300 annotated genomes. This process was permutated three times, each time spiking each variant of the set of 300 ClinVar variants into different genomes out of the 300. The outcome is a simulation of 900 artificial patients with 300 unique “genetic diseases” (Additional file 1: Table S2), each with a single mutation in one of 300 genes. This ensured that each disease appeared three times in three different genomes.

Phenotypic information for each spiked ClinVar gene variant was retrieved from the HPO (Human Phenotype Ontology) database [46]. Additionally, to evaluate the robustness of EvORanker, we conducted validation through three independent spike shuffles (Additional file 1: Table S2, Additional file 2: Fig. S7).

### Genomics and variant-based prioritization of the patients’ exome to map the patient candidate genes

Whole-exome sequencing was performed on the patients described in this study using the Truseq Capture Exome Kit (Illumina®). The captured and the amplified libraries were sequenced on the Illumina Nextseq500 platform according to the manufacturer’s protocol. Briefly, paired-end sequences were obtained at a read length of 150 bps. Sequence reads were then aligned to the reference human genome (hg19) using BWA aligner [47]. Alignments then underwent preprocessing steps by PCR duplicate removal, base quality recalibration, and realignment around indels. The variants were finally called by GATK (Genome Analysis Toolkit) [48] and annotated by ANNOVAR [41]. For both the patient exomes and simulated genomes, we excluded intronic, untranslated region (UTR), and ncRNA variants. Nonsense, frameshift, nonsynonymous, and splice-site variants were prioritized by excluding frequent variants based on their minor allele frequency in gnomAD and AF_popmax [42] and Istishari hospital’s in-house exome database (Table 1). In addition, variants predicted to be benign by variant effect predictor tools such as PolyPhen-2 [5] and REVEL [6] were excluded from the analysis (Table 1) [49]. For the patient exomes, copy number variations were called from the exome data using XHMM software [50]. In this study, the genes that passed the variant filtering criteria are referred to as the *patient candidate genes*. Co-segregation analysis was performed by Sanger sequencing on an ABI 3130 Genetic Analyzer (Applied Biosystems). Primers used for validation and family segregation of the *DLGAP2* variant are as follows: Forward: CGGTAGAGACTGGGAGGATG and Reverse: ACTTACCTGACAAAACACACACA. Primers used for validation and family segregation of the *LPCAT3* variant are as follows: Forward*:* CGCATAGGGGTGACATGGTA and Reverse*:* TATGCATTTTGACGGGCCTG.

### Ranking the patient candidate genes according to their association with each patient’s disease-phenotypes

In order to retrieve a list of genes already reported to be associated with each patient’s clinical condition, we encoded the abnormalities reported in each patient’s medical report (Additional file 1: Table S1) to standard HPO terms [46]. The combination of the patient HPO terms was then input into the OntologySimilarity package in R, a semantic similarity-based tool [51], and used to identify and rank the genes listed in the HPO database based on their associations with the queried HPO terms. According to the recommended parameters in the tool’s documentation [51], the semantic similarity score was calculated using Lin’s definition of semantic similarity in combination with the “best-match-average” approach. Additional semantic similarity measures, such as the product measure based on Resnik’s similarity expression, were also evaluated, yielding results that were nearly identical (Additional file 2: Fig. S1). The output is a ranked list of the 4900 genes listed in the HPO database (as of January 2023) scored between zero and one (termed HPO-ranked genes). This score is based on the degree of similarity between the patient’s set HPO terms and the HPO terms annotated to each gene in the HPO database. The higher-scoring genes, referred to as *phenotype-related genes* in this study, are defined as genes that exhibit stronger associations with the queried phenotypes.

Defining an exact threshold for the output of OntologySimilarity [51] is challenging. In most cases, it is hard to point to a clear threshold that above which the genes are “phenotype-related.” Furthermore, such a potential threshold is highly dependent on the user-defined HPO terms as HPOs are variable in their level of complexity and how well they are defined or studied. As such in our analysis, the genes were ranked and ordered based on their association with the set of input phenotypes, ranging from highly associated to not associated (top to bottom). We utilized a one-sided Kolmogorov-Smirnov test to assess the significance of a skewed distribution of co-evolved (or interacting genes) towards the upper end of the ranked genes (see below).

### Building the EvORanker algorithm

The main goal of EvORanker is to establish a link between the *patient candidate genes* and the patient’s phenotype. This link was evaluated based on two different sources of data on known and predicted gene functional interactions:, clade-wise phylogenetic profiling, and the STRING database [33]. Our working hypothesis is that of the *patient candidate genes*, the disease-causing gene would be functionally linked to the genes that cause similar phenotypes to those of the patient (e.g., the disease-causing gene in a patient with ciliopathy would show significant co-evolution/co-expression/interaction with cilia genes). We used the 109-patient exome and 900-simulated datasets to tune the parameters of the algorithm using exclusively phylogenetic profiling (PP). Subsequently, we compared and eventually integrated the PP-based analysis with the STRING-based analysis to maximize the performance of EvORanker.

### Clade-based phylogenetic profiling

The normalized phylogenetic profiling (NPP) matrix was constructed as previously described [24]. Briefly, a matrix of BLASTP scores for all human genes against the genomes of 1,028 eukaryotic species was constructed. First, the bitscore of each best BLAST hit was normalized by the bitscore of the query protein self-hit. Then log2 transformation of the normalized bitscore was applied. Finally, to avoid any biases due to phylogenetic distance, the conservation score was scaled for each species to their overall distribution by transforming the values in the column (corresponding to a species) into *z*-scores.

#### Clade-based analysis

To have a comprehensive mapping of protein-correlated evolution, we used 16 representative clades spanning the eukaryotic tree as previously described [24, 30]. In addition to including all eukaryotes, the following clades were used: *Chordata*, *Ecdysozoa*, *Platyhelminthes*, *Alveolates*, *Stramenopiles*, *Fungi*, *Viridiplantae*, *Mammalia*, *Archelosuria*, *Arthropoda*, *Nematoda*, *Basidiomycota*, *Ascomycota*, *Fungi incertae sedis*, *Liliopsida*, and *Eudicotyledons* [24]. These 16 representative clades spanning the eukaryotic tree show wide coverage (span most of the eukaryotic tree), mutual exclusivity (preferring non-nested clades), and uniformness (similar depth in the tree) in clade types (Additional file 2: Fig. S2) [24].

#### Retrieving coevolving genes for each patient candidate gene

The degree of co-evolution between two genes was evaluated using the Pearson correlation coefficient between their respective rows in the NPP matrix. As we demonstrated before [24], for each *patient candidate gene*, we selected the genes with the top 100 correlation coefficients in each clade and ranked them from 1 to 100 according to the correlation coefficient in each clade where the gene is found to have an ortholog.

### Using the Kolmogorov-Smirnov test to prioritize patient candidate genes based on phylogenetic profiling

Per patient exome/simulated genome, we analyzed each of the *patient candidate genes* separately. For each of these genes, we determined whether the genes that co-evolve with it were associated with the patient’s phenotype (i.e., the co-evolved genes were significantly enriched towards the *phenotype-related genes*). For that, we examined the ranking of the coevolving genes in the list of the *HPO-ranked genes* using a one-tailed, two-sample Kolmogorov-Smirnov (K-S) test [52].

The K-S test is used to test whether two samples come from the same distribution. The K-S ***D*** statistic quantifies the distance between the empirical cumulative distribution function (ECDF) of the sample and the cumulative distribution function of the reference distribution. Let *i* denote the co-evolving genes and *j* denote the *ranked HPO genes*.

The null hypothesis: H0:*Fi(x)* ≥ *Fj(x)*

The alternative hypothesis: H1:*Fi(x)* < *Fj(x)*

The D statistic: ***D-*** = max_*x*_{*Fj*(*x*)-*Fi*(*x*)} where *Fj* is the ECDF of *j* and similarity for *Fi*

The H1 hypothesis for the one-sided K-S test is that the cumulative distribution function of the ranking of the coevolving genes is enriched within the higher-scoring side of *HPO-ranked genes (the phenotype-related genes)*. A *p*-value was computed using the ks.test function in the stats package in R [53]. The *patient candidate genes* were finally ranked by the resulting K-S test *p*-value (from more significant to less significant).

### Tuning the parameters of the EvORanker phylogenetic profiling-based analysis

We evaluated the performance phylogenetic profiling-based analysis to identify the “true” disease-causing gene using the 109-patient exome and the 900-simulated databases. We examined different parameters and cutoff values using phylogenetic profiling. We compared different cutoff values of the ranked coevolving genes (top 10, 25, 50, 75, 100) with each *patient candidate gene*. In both datasets, a threshold of the top 50 coevolved genes yielded slightly better accuracy in ranking the “true” gene in comparison to the other patient candidate genes (Additional file 2: Fig. S3), which we used for the rest of the analysis.

### Applying the Kolmogorov-Smirnov test using STRING-interacting genes

In addition to phylogenetic profiling, other known and predicted functionally associated genes (protein-protein interactions, text mining, and co-expression) were retrieved from the STRING database [33]. STRING uses a scoring system that reflects the evidence of predicted interactions. We included interactions with a combined score of at least 0.5, which corresponds to a medium-confidence network. For each patient exome/simulated genome in the datasets, we applied the K-S test. The analysis was done for each *patient candidate gene*, with the STRING-interacting genes similarly as described above. We examined whether a substantial portion of string-interacting genes were also linked to phenotypes resembling those found in the patient.

### The final EvORanker gene prediction scoring system

The two *p*-values obtained from each K-S test using phylogenetic profiling and STRING were finally combined by Fisher’s combined probability test [54, 55] (Eq. 1) which is the final EvORanker scoring system. Additionally, we assessed Simes’ method for combining the *p*-values, which produced similar results (Additional file 2: Fig. S1). The Fisher’s combined probability test was computed using the combine.test function in the survcomp package in R [56].

1 $${X}_{2k}^{2}\sim -2\sum_{i=1}^{k}{\text{log}}\left({p}_{i}\right)$$

### Applying EvORanker on genes with knockout phenotypes in mice that lack corresponding human annotation

A list of 6395 human genes with mouse knockout phenotypes but not yet associated with a phenotype in humans was compiled from Jackson laboratory’s Mouse Genome Informatics (MGI) [57], (downloaded, March 1, 2023). The knockout mouse gene phenotype terms were then mapped to human HPO terms using uPheno ontology inter-ontology closest matches obtained from the OBO Phenotype Ontology Github repository [58] ending up with 6260 genes with mapped HPO terms. Then, for each gene, the corresponding HPO terms and a list of randomly sampled genes were input to EvORanker. The same data was input to Phenolyzer [59] for comparison. We were unable to compare to other tools (e.g., ExomeWalker or PHIVE) due to the impracticability of simulating 6,260 × 100 variants in 6,260.vcf files as input.

### Tool comparison

We compared EvORanker to the gene prioritization stage (second stage) of ExomeWalker and PHIVE algorithms [3, 8]. We used the 109-patient exome and 900-simulated benchmarking datasets to compare the tools with the same input HPO terms and *patient candidate genes*. We omitted one exome from the exome dataset where a large deletion was identified containing the *NPRL3* gene, leaving us with 108 exomes. We used.vcf files for each of the 108 patient exomes and the 900 genomes as input for Exomiser which includes ExomeWalker [8] and PHIVE [3]. Additionally, we created.yml files containing the same HPO terms as input for each patient exome and simulated genome.

### In vitro splicing analysis

In vitro splicing, minigene assays were carried out as previously described [60, 61]. Briefly, the genomic sequence at chr8:1626251-1627026 (hg19) which includes exon 9 (417 bp) plus 128 and 231 nucleotides from the 5′ and 3′ flanking sequences, respectively, of *DLGAP2* (NM_001346810) was PCR amplified from a DNA sample homozygous (II-3, Fig. 10A) and wildtype (II-2, Fig. 10A) for the c.2702 A > T variant using gene-specific primers designed with embedded XhoI and BamHI restriction enzyme recognition sites. After digestion, the PCR fragments were ligated into a pre-constructed pET01 Exontrap vector (MoBiTec, Goettingen, Germany). Selected colonies were then sequenced to confirm the proper orientation of the cloned fragment and identify both wild-type and variant colonies. Subsequently, the variant and wild-type minigenes were transfected into HEK293 cells in triplicate, followed by total RNA extraction 48 h post-transfection, using the Quick-RNA MiniPrep Plus kit (ZYMO Research). cDNA was then synthesized using the qScript Flex cDNA synthesis kit (Quanta Biosciences) with a specific primer to the 3′ native exon of the pET01 Exontrap vector. Following PCR amplification, the products were then visualized on a 1.5% agarose gel and were later extracted and then Sanger sequenced. The primer sequences used for the PCR amplification (XhoI + BamHI) are Forward: AAA-CTCGAG-AACACTACCTGCCCTTGAGC, and Reverse: AAA-GGATCC-ACTTACCTGACAAAACACACACA.

### Data analysis and figure creation

All data in this study were analyzed using R software [53]. The EvORanker web interface was created using the R Shiny package [62]. The majority of the figures were created using R software. Figures 9D and 10D were created using Cytoscape v3.9.1 [63].

## Results

### Overview

In this work, we developed EvORanker, a phylogenetic profiling-based algorithm, to identify disease-causing genes. To optimize and evaluate the performance of EvORanker, we employed three different approaches: (1) analyzing a private cohort of well phenotypically characterized patients with rare diseases; (2) simulating a dataset of 900 patients with 300 unique “genetic diseases”—by spiking disease-causing mutations into real genomes; (3) evaluating EvORanker’s ability to identify human disease candidate genes using genes with knockout phenotypes in mice that lack corresponding human annotation. We demonstrate the contribution of clade-based phylogenetic profiling (PP) to the improved prediction of the disease-causing gene. This unbiased approach was compared and integrated with gene interaction data obtained from the STRING database [33]. To evaluate the potential for bias in disease-gene prediction, we compared well-annotated genes to recently published ones. Finally, EvORanker was compared to other gene-based prioritization tools and applied to two unresolved exomes to demonstrate its efficacy in disease gene discovery.

### Benchmarking EvORanker using an exome-patient dataset

We analyzed an in-house database of 109 patient exomes with a genetic diagnosis. The patients suffer from various rare hereditary diseases, exhibiting diverse phenotype groups (e.g., skeletal, immunological, neurological, and metabolic diseases) (Fig. 2A). The dataset included 91 recessive and 18 dominant gene variants that explained the patients’ phenotype (Additional file 1: Table S1). All these variants are reported to be pathogenic/likely pathogenic in the ClinVar database [38] and co-segregated with the phenotype in each corresponding family. The dataset includes 108 unique known disease genes (the *CLCN1* gene appears twice, once as autosomal recessive and once as autosomal dominant). For each patient in the exome dataset, we encoded each of the clinical abnormalities found in the patient’s medical record into Human Phenotype Ontology (HPO) [46] terms (Additional file 1: Table S1).

### Benchmarking EvORanker using a simulated dataset

Next, we aimed to assess our ability to identify disease-causing mutations in simulated data. Simulating genetic diseases can be achieved by introducing pathogenic mutations into genomic data from an unaffected individual. To accomplish this, we utilized 300 unaffected genomes sourced from the 1000 Genome Project [39] as a benchmark for our evaluations. To introduce pathogenicity, we randomly integrated 300 distinct pathogenic/likely pathogenic variants from the ClinVar database [38] into the annotated genomes. These ClinVar variants are associated with genes showcasing diverse phenotypes including complex neoplastic disorders (Fig. 2B). Of these variants, 181 followed an autosomal or X-linked recessive mode of inheritance, while 119 variants followed a dominant inheritance pattern (Additional file 1: Table S2). Phenotypic information for each spiked ClinVar gene variant was obtained from the HPO database (Additional file 1: Table S2) and was assigned to the respective “patient.” We conducted this process thrice, simulating a total of 900 artificial patients with 300 different genetic diseases. Each pathogenic mutation was inserted into three distinct genomes.

#### Ranking genes based on each patient’s set of phenotypes

Using each set of patient HPO terms, we calculated the semantic similarity score [51] (see the “Methods” section) for each gene in the HPO database [46]. The output is a list of genes scored from lower association to higher association with the patient’s set of HPO terms (which we term the phenotype-related genes).

#### Retrieving the patient’s candidate genes

We applied our routine variant filtering criteria [35, 36] to the annotated variants for each of the 109 exomes and simulated genomes (Table 1). After variant filtering, each exome/genome contained gene variants that are considered to be pathogenic and predicted to affect protein function (we term the genes in which these variants were observed as *patient candidate genes*). In autosomal and X-linked recessive cases, each patient harbored 11–80 homozygous/hemizygous or compound heterozygous deleterious variants, while 80–170 heterozygous/hemizygous deleterious variants were observed in autosomal and X-linked dominant cases (Additional file 2: Fig. S4). We confirmed that all the “true” causative variants passed the filtering criteria and remained within the gene variant list for each patient exome.

#### Using multi-clade phylogenetic profiling to rank the patient’s candidate genes according to the patient’s phenotype

Our working hypothesis is that out of all the patient candidate genes, the one responsible for the disease will be associated (e.g., co-evolved) with other genes that are known to be associated with the disease (phenotype-related gene). For each *patient candidate gene*, we obtained a list of 50 co-evolved genes that exhibit a strong correlation based on global and local co-evolution signatures across 1028 eukaryotic species (details in the “Methods” section) [24]. For each *patient candidate gene*, we retrieved the top co-evolving genes in 16 clades (*Chordata*, *Ecdysozoa*, *Platyhelminthes*, *Alveolates*, *Stramenopiles*, *Fungi*, *Viridiplantae*, *Mammalia*, *Archelosuria*, *Arthropoda*, *Nematoda*, *Basidiomycota*, *Ascomycota*, *Fungi incertae sedis*, *Liliopsida*, and *Eudicotyledons*) (Additional file 2: Fig. S2). The output per *patient candidate gene* is a table of genes that are strongly co-evolved with it in each clade in addition to all Eukaryotes.

**Fig. 3:**
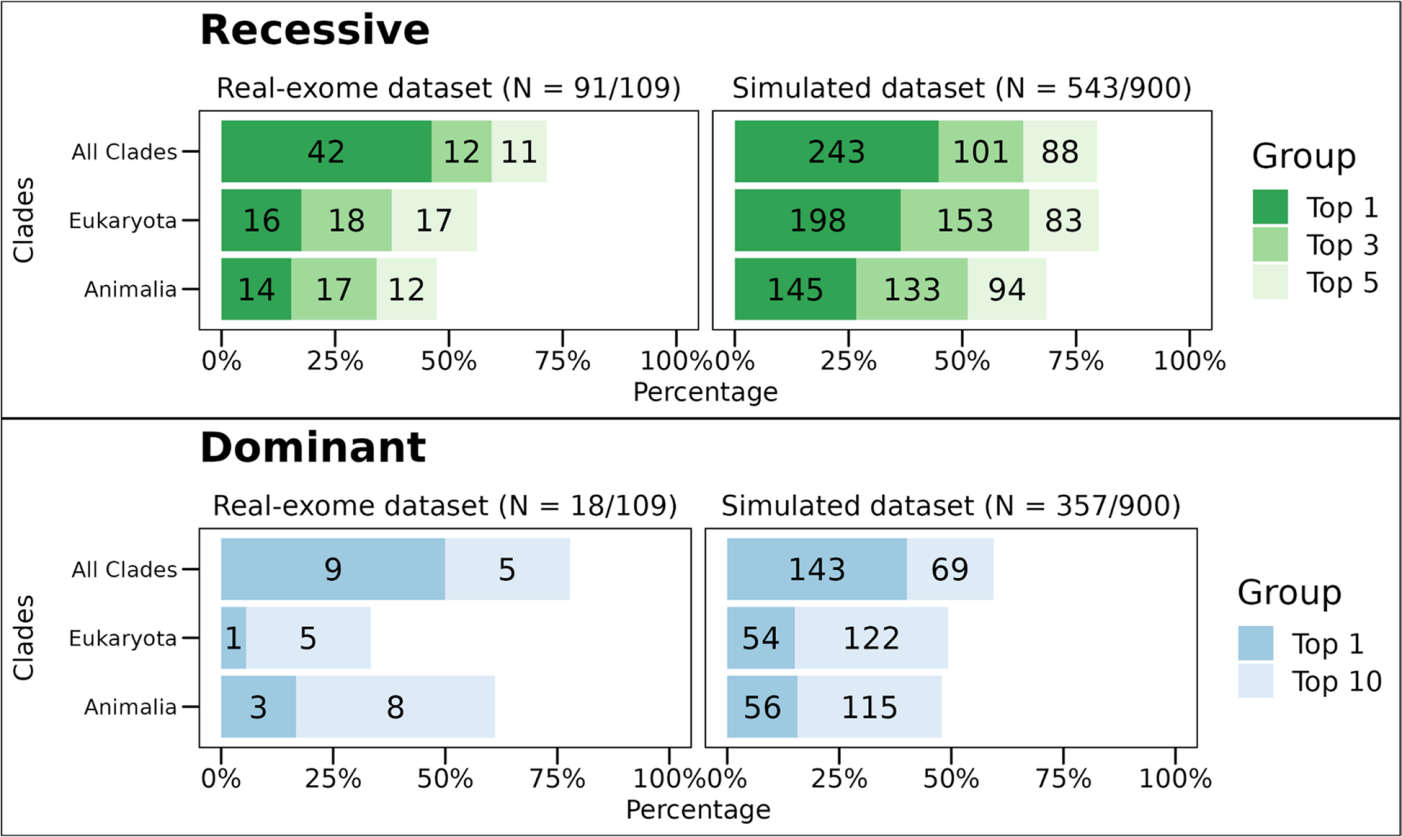
Using clades improves the performance of EvORanker phylogenetic profiling-based analysis. For each patient candidate gene list in the 109-patient exome and the 900-simulated genomes datasets (300 unique genetic disorders), we compared the accuracy of the phylogenetic profiling-based algorithm by retrieving the top 50 coevolved genes with each *patient candidate gene* across all Eukaryotes versus: (1) using all 16 clades where the query gene has an ortholog in addition to Eukaryotes. (2) Across only Animalia clades (*Chordata*, *Mammalia*, *Archelosauria*, *Ecdysozoa*, *Nematoda*, *Arthropoda*, and *Platyhelminthes*). Performance was measured by examining the ranking of the “true” disease-causing gene relative to the other patient candidate genes. The upper bar plot shows results for the autosomal and X-linked recessive cases for the real-exome dataset (left) and the simulated dataset (right). The simulated dataset contains 181 unique recessive cases and 119 unique dominant cases. The results present a compilation of three separate independent shuffles totaling 900 simulations. The lower bar plot shows results for the autosomal and X-linked dominant cases. The *y*-axis indicates the tested clades, and the *x*-axis indicates the percentage of cases where the “true” disease gene was ranked at the top or within the top 3 or top 5 genes relative to the other candidate genes in recessive cases. In dominant cases, the percentage is for the “true” gene being ranked at the top or within the top 10 genes. Overall, the best performance of ranking the “true” causative gene was achieved by merging together the co-evolving genes within all clades (the 16 clades in addition to all Eukaryota) in both datasets

To determine which of the *patient candidate genes* is most likely linked to the patient’s disease phenotype, we evaluated the intersection between the co-evolved genes and the phenotype-ranked genes using a one-sided *Kolmogorov-Smirnov* (K-S) test (Fig. 1). A significant *p*-value is obtained if the co-evolving genes rank high within the *phenotype-related genes*. For each patient exome/simulated genome in our dataset, we ranked the *patient candidate genes* based on the resulting *p*-value, with the most significant *p*-value ranked first. By analyzing the co-evolved genes across the 16 clades in addition to all Eukaryotes, the “true” disease-causing gene was ranked as the top gene in 46% of the autosomal and X-linked recessive cases and within the top 5 in 72% (Fig. 3). In autosomal and X-linked dominant cases, the “true” gene was ranked as the top gene in 50% of the cases and within the top 10 genes in 78%. These results surpass those obtained from using only the co-evolving genes across Eukaryotes or within the Animalia clades (*Chordata*, *Mammalia*, *Archelosauria*, *Ecdysozoa*, *Nematoda*, *Arthropoda*, and *Platyhelminthes*) (Fig. 3). This indicates the added value of incorporating all 16 clades in the analysis. The same analysis was applied on the simulated genomes, yielding results consistent with those obtained from the real exome dataset (Fig. 3).

#### Phylogenetic profiling analysis in different evolutionary scales improves the prediction of the disease-causing gene

**Fig. 4:**
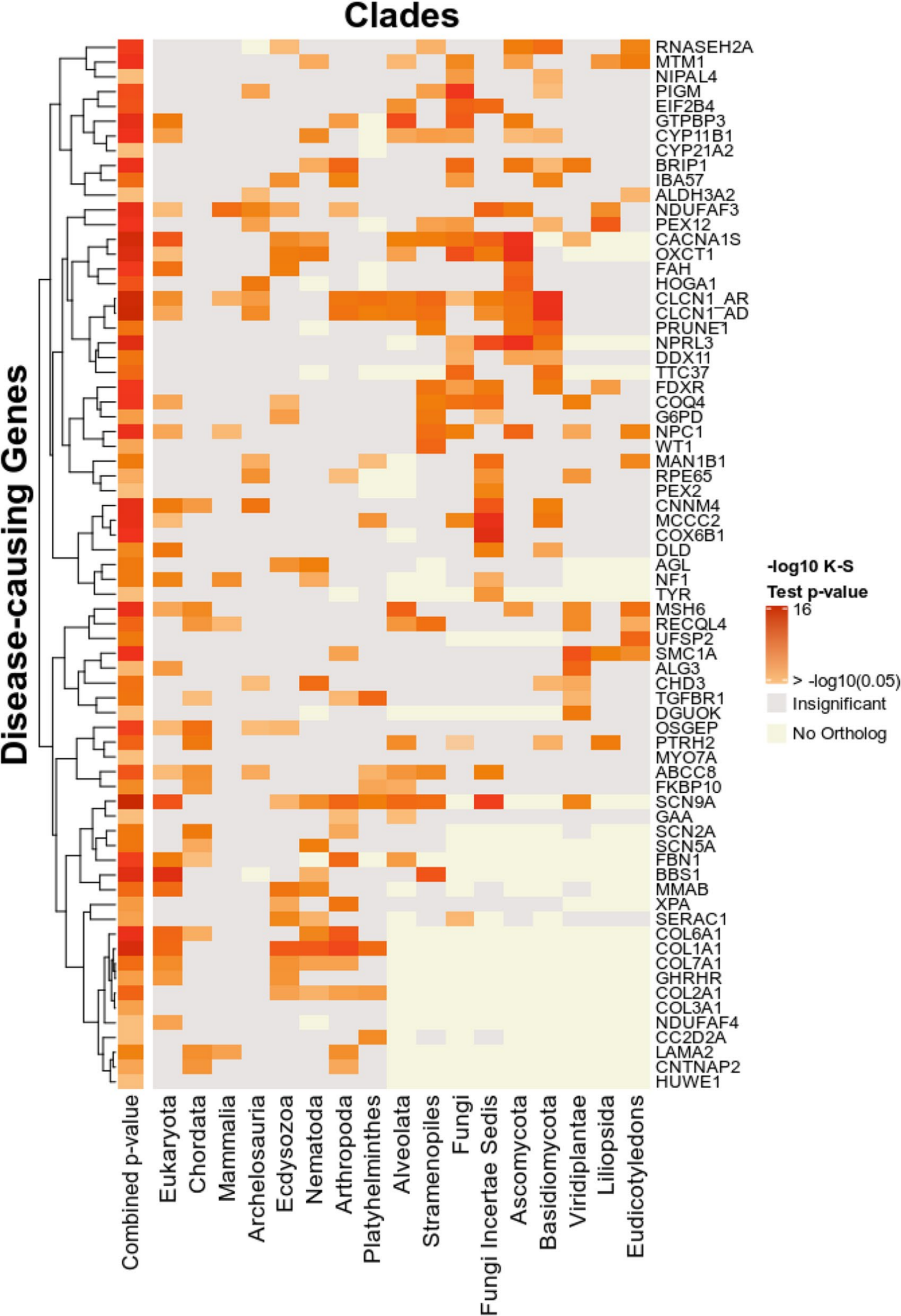
Each of the 16 clades in addition to *Eukaryota* contributes to the correct identification of the disease-causing gene. Each column in the heatmap represents a clade while each row represents the “true” disease-causing gene in a patient exome from the 109-exome patient dataset. Only the genes that achieved an overall significant K-S test *p*-value (< 0.05) using the co-evolution analysis are displayed (71 cases). Each entry in the heatmap is colored by the -log 10 of the K-S test *p*-value that was run on each clade separately. The entries colored in red represent the significant *p*-values (> -log10(0.05)). Light grey entries indicate non-significant *p*-values. Entries, where the gene is not found to have an ortholog in a certain clade, are colored off-white. The rows are clustered according to the *p*-values. The column on the left indicates the combined -log10 of the *p*-value obtained by running the K-S test after merging together the coevolving genes across the clades. In four cases (*HUWE1*, *COL3A1*, *MYO7A*, and *CYP21A2*), a significant *p*-value was obtained by none of the clades, but a significant combined *p*-value was still achieved by merging the co-evolving genes from all the clades

We then aimed to assess the contribution of each of the 16 clades, in addition to all *Eukaryota*, towards the prediction of the “true” disease-causing gene. Using the 109 patient exomes, this was accomplished by focusing on the genes that obtained a significant *p*-value (< 0.05) through co-evolution analysis totaling 71 identified genes. We applied the K-S test to these 71 genes using the co-evolving genes within each clade. Results showed that each clade outperformed others in at least one case, thus highlighting the importance of combining information from different clades to enhance the performance of EvORanker (Fig. 4). Interestingly, the *Fungi Incertae Sedis* clade outperformed other clades in 14% (10/71) of the cases, followed by *Chordata*, *Ascomycota*, *Arthropoda*, and *Eukaryota*, each outperforming others in 10% of the cases (Additional file 2: Fig. S5). Taken together, these results emphasize that clades differentially specialize in detecting functional interactions in different pathways [24, 29, 30].

#### Phylogenetic profiling is complementary to other existing omics datasets

**Fig. 5:**
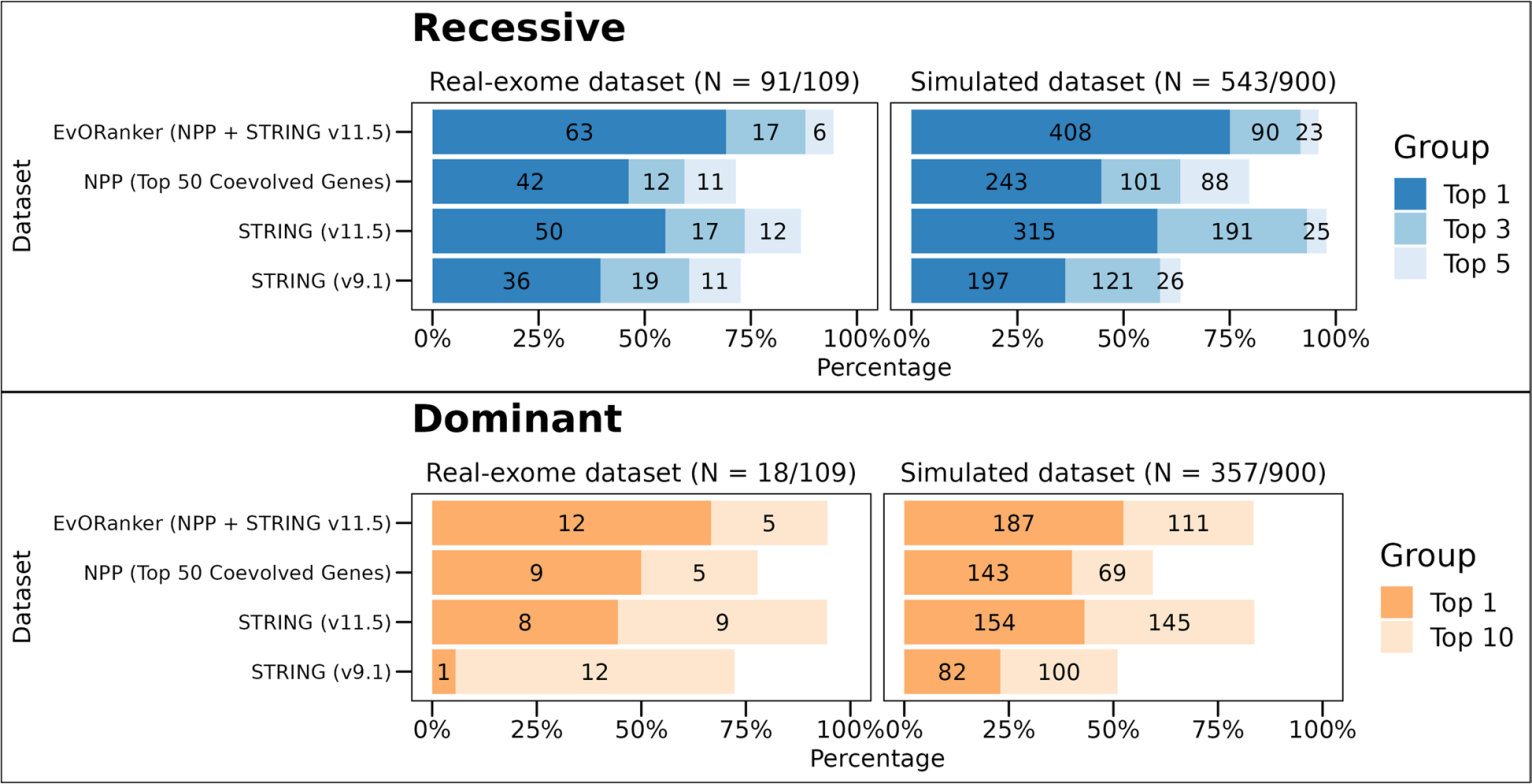
Comparative performance of NPP, STRING, and EvORanker using the 109-patient exome and the simulated datasets. The performance of each dataset was measured by examining the ranking of the “true” disease-causing gene relative to the other genes in each exome/genome in both datasets. The upper bar plot shows results for the autosomal and X-linked recessive cases for the real-exome dataset (left) and the simulated dataset (right), The simulated dataset contains 181 unique recessive cases and 119 unique dominant cases. The results present a compilation of three separate independent shuffles totaling 900 simulations. The lower bar plot shows results for the autosomal and X-linked dominant cases. The *y*-axis indicates the tested datasets: NPP (using the top 50 coevolved genes), STRING versions 9.1, 11.5, and EvORanker (combining NPP and the newer version of STRING). The *x*-axis indicates the percentage of cases where the “true” disease gene was ranked at the top, or within the top 3 or top 5 genes relative to the other candidate genes in recessive cases. In dominant cases, the percentage is for the “true” gene being ranked at the top or within the top 10 genes. Overall, the best performance was achieved using the combined approach (EvORanker) in both datasets

NPP represents an unbiased approach that can annotate gene function independently of the literature. We sought to evaluate whether clade-wise NPP could identify disease-associated genes that are overlooked by other existing omics. For that, we chose to use the STRING database since it integrates information on protein associations from multiple sources, including interaction experiments, known complexes and pathways, scientific literature, co-expression studies, and conserved genomic context [33]. We conducted a comparison between the NPP and STRING-based analysis [33] using both the patient exome and simulated datasets (Fig. 5). NPP outperformed STRING in 29/109 (27%) of the cases, whereas STRING outperformed NPP in 50/109 (46%) of the cases (Fig. 5, Additional file 2: Fig. S6).

Considering the presence of complementarity of co-evolution and the STRING-based analysis, we integrated the two datasets by combining their respective *p*-values using Fisher’s combined probability test [54]. This combined scoring system, which we termed EvORanker, yielded the highest accuracy in comparison to each dataset alone (Fig. 5). Using the exome dataset, we showed that integrating NPP and STRING improved the results by 43% compared to NPP alone and by 30% compared to STRING alone (Additional file 2: Fig. S6). Overall, in autosomal and X-linked recessive cases, EvORanker ranked the “true” disease-causing gene as the top gene in 63/91 (69%) and within the top 5 genes in 86/91 (95%) cases (Fig. 5). In autosomal and X-linked dominant cases, the “true” gene was ranked as the top gene in 12/18 (67%) cases and among the top 10 genes in 17/18 (95%) (Fig. 5). On the other hand, the “true” disease genes did not achieve high scores in a total of 6/109 (5.5%) of the exomes (within the top 5 for recessive diseases and within the top 10 for dominant diseases); 5/91 recessive cases, and 1/18 dominant cases (Additional file 2: Fig. S6).

We observed similar trends when analyzing the simulated dataset, providing further validation and affirming the consistency of our findings (Fig. 5). In autosomal and X-linked recessive cases within the simulated dataset, EvORanker ranked the “true” disease-causing gene as the top gene in 75% of cases and within the top 5 in 96% of cases. Conversely, for autosomal and X-linked dominant cases, the “true” gene held the top position in 55% of cases and was within the top 10 in 85% of cases. This parallel in results strongly underscores the robustness of EvORanker across both real and simulated datasets. Furthermore, to validate the stability of our method, we conducted three independent spike shuffles, consistently yielding coherent and reliable results (Additional file 2: Fig. S7).

### Performance of phylogenetic profiling versus STRING on new gene entries (2020–2022)

**Fig. 6:**
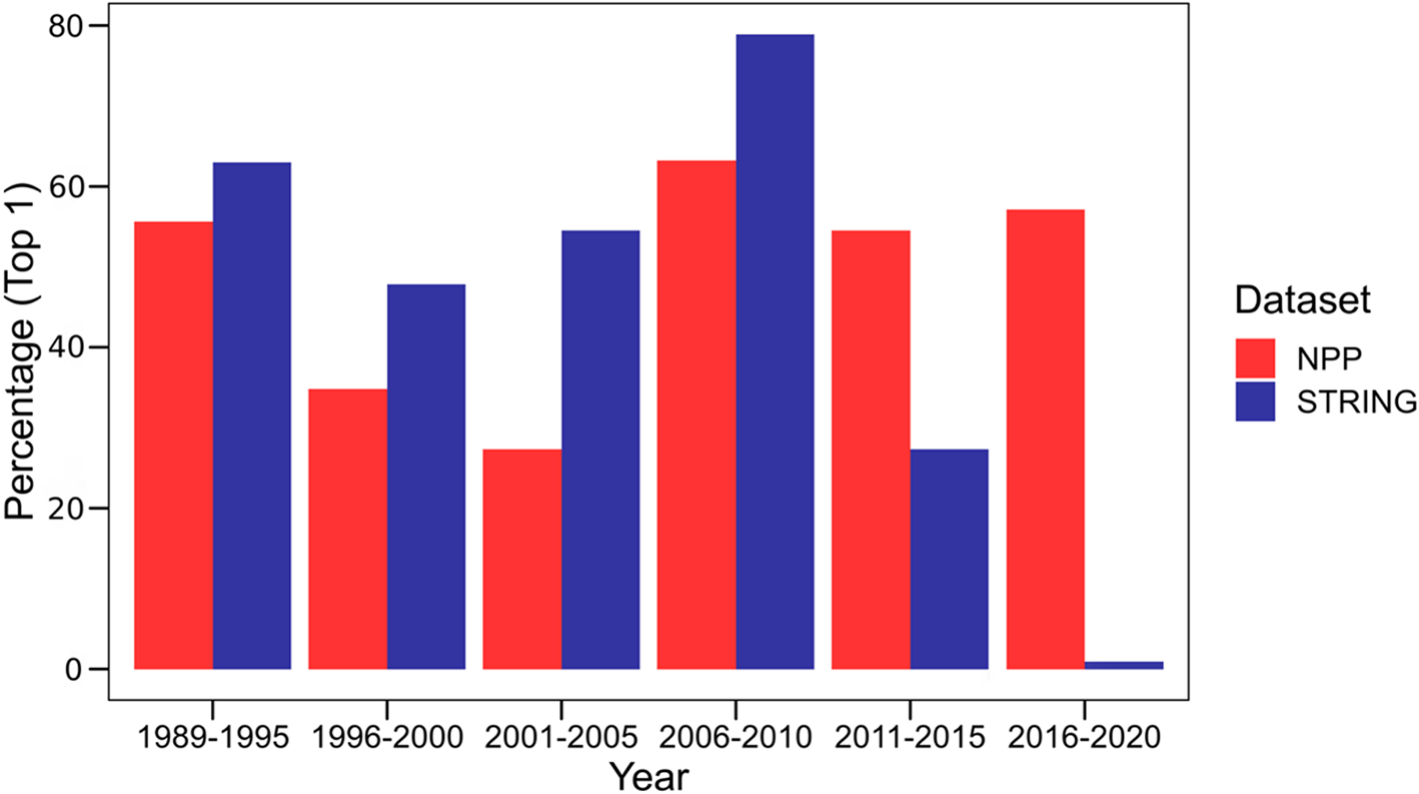
The effect of years elapsed on the performance of NPP versus STRING, using the 109-patient exome dataset. The *x*-axis indicates the calendar years (divided into 5-year windows) in which a gene was described to be associated with a disease phenotype. The *y*-axis indicates the percentage of “true” disease genes that ranked at the top (top 1) relative to the other *patient candidate* using NPP (red bars) or STRING (blue bars)

As STRING is based on publicly available data, it is suited to identify well-researched genes. We hypothesized that STRING performance would be better the more information it has accrued over time and that our unbiased PP approach would have a particular advantage for genes that have not been extensively characterized. To test this hypothesis, we compared the performance of STRING version v.11.5 [33] with that of the older version STRING v.9.1 [64] (Fig. 5). We found that the performance of the newer version was indeed better than that of the older version. Furthermore, the performance of STRING v11.5 decreased dramatically for genes that only recently became associated with disease. For example, the performance of STRING in ranking the “true” disease gene within the top 5 is around 85% for genes identified by the end of 2015 compared to 29% for genes identified between 2016–2020 (Fig. 6, Additional file 2: Fig. S8).

**Fig. 7:**
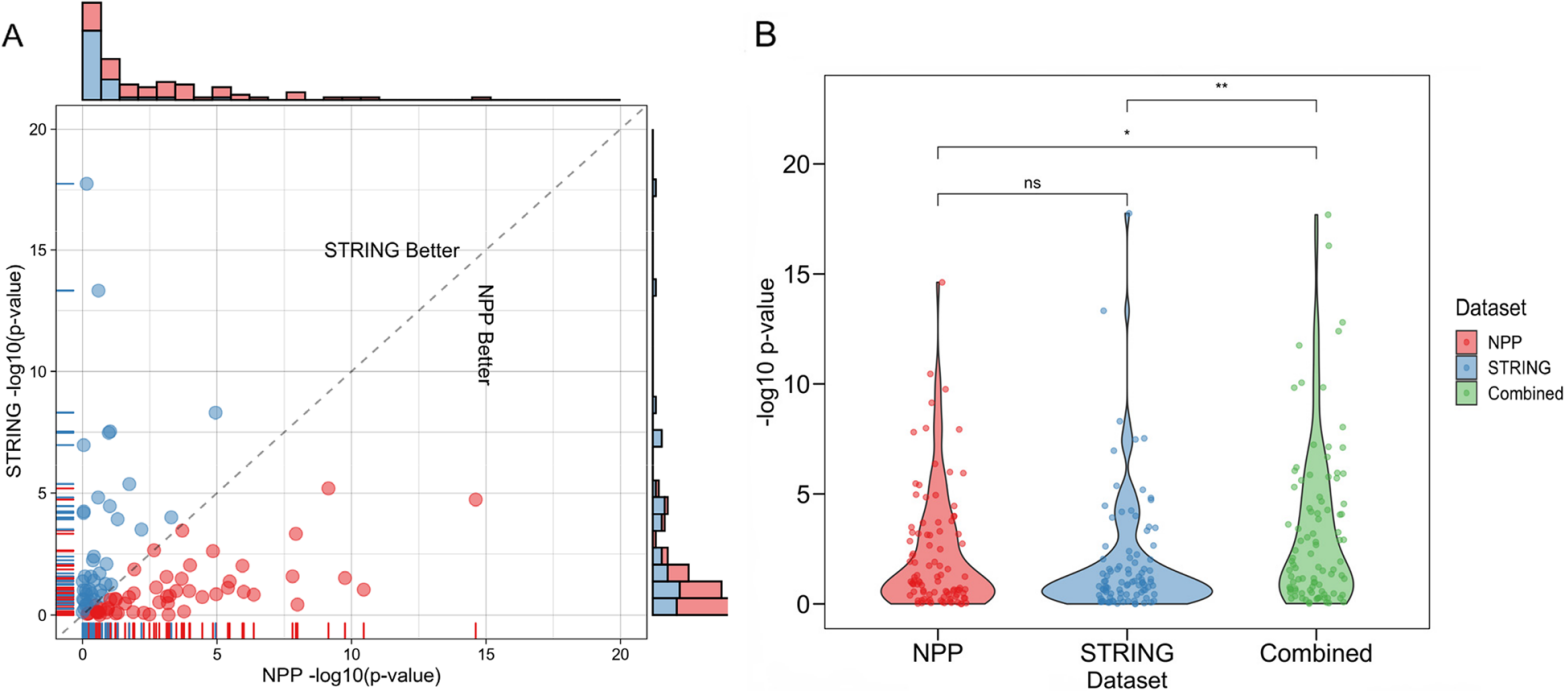
Comparison of NPP versus STRING for genes with recent (2020–2022) annotation. **A** The *x*-axis indicates -log(10) *p*-values obtained from running the K-S test using NPP. The *y*-axis indicates -log(10) *p*-values obtained from running the K-S test using the STRING dataset. The red dots represent the genes where NPP performed better than STRING, while the blue dots indicate the opposite. The marginal histogram indicates the distribution of the -log(10) *p*-values of both datasets. The correlation score between the two datasets is 0.046, suggesting that the two datasets exhibit a complex relationship, where a subset of the data displays complementarity, while another subset shows correlation. **B** Density distribution of the -log(10) *p*-values obtained from the K-S test using the NPP, STRING, and both (combined). Significance was calculated using the Wilcoxon test (*p-value < 0.05, **p-value < 0.01; ns, nonsignificant). Combining NPP and STRING achieved significantly more significant results that either approach alone

We then evaluated the performance of NPP and STRING on newly discovered or recently published genes. We retrieved a list of 94 new gene entries that were added to the most recent version of the HPO database (2022) compared to an older version (2020) (Additional file 1: Table S3). We then applied the K-S test separately using NPP and STRING and the HPO terms associated with each gene as input. We found that for those genes newly associated with human phenotypes, the K-S test yielded significant *p*-values using NPP in 45% of the genes compared to 38% using STRING (Fig. 7A, B). These results emphasize the success of phylogenetic profiling in predicting the phenotype associations of newly discovered or less studied genes and highlight the complementarity observed when comparing these two datasets.

### Performance of EvORanker on genes with knockout phenotypes in mice that lack corresponding human annotation

We aimed to assess EvORanker’s capability to identify disease candidate genes that lack a known phenotype association in humans but possess mouse orthologs linked to phenotypes. Specifically, we aimed to identify human genes without established phenotype links, yet having a corresponding mouse ortholog with a phenotype association. These genes were considered as the “true” disease gene candidates for the purpose of this evaluation. We compiled a list of 6260 human ortholog genes with mouse knockout phenotypes, yet not associated with a phenotype in humans. For each of these genes, we input a set of HPOs mapped from the respective mouse knockout phenotypes. The goal was to evaluate EvORanker’s ability to correctly pinpoint the “true” disease gene candidate in comparison to 100 randomly sampled human genes. The same dataset was used as input for Phenolyzer [59] for comparative analysis.

**Fig. 8:**
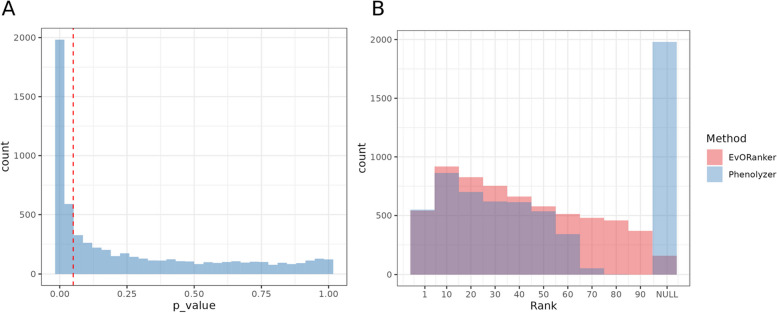
EvORanker’s performance in identifying candidate disease genes using mouse knockout genes without corresponding human annotation. **A** The graph shows the percentage of genes with mouse knockout phenotypes that were tested for significant *p*-values using EvORanker. Out of 6260 genes, 41% showed significant *p*-values. **B** Comparison of EvORanker and Phenolyzer [49] in identifying true disease gene candidates. The graph shows the count of genes with mouse knockout phenotypes and their respective ranking, each in comparison to 100 randomly sampled genes by EvORanker and Phenolyzer. Among the tested genes, 16% were ranked in the top 10 by both tools

EvORanker yielded significant *p*-values for 41% of the tested genes (Fig. 8A). Moreover, both EvORanker and Phenolyzer ranked the “true gene” among the top 10 in 16% of the cases (Fig. 8B). Notably, EvORanker identified genes that Phenolyzer failed to identify, and vice versa, highlighting the complementarity of the tools (Additional file 2: Fig. S9).

### Tool comparison

**Fig. 9:**
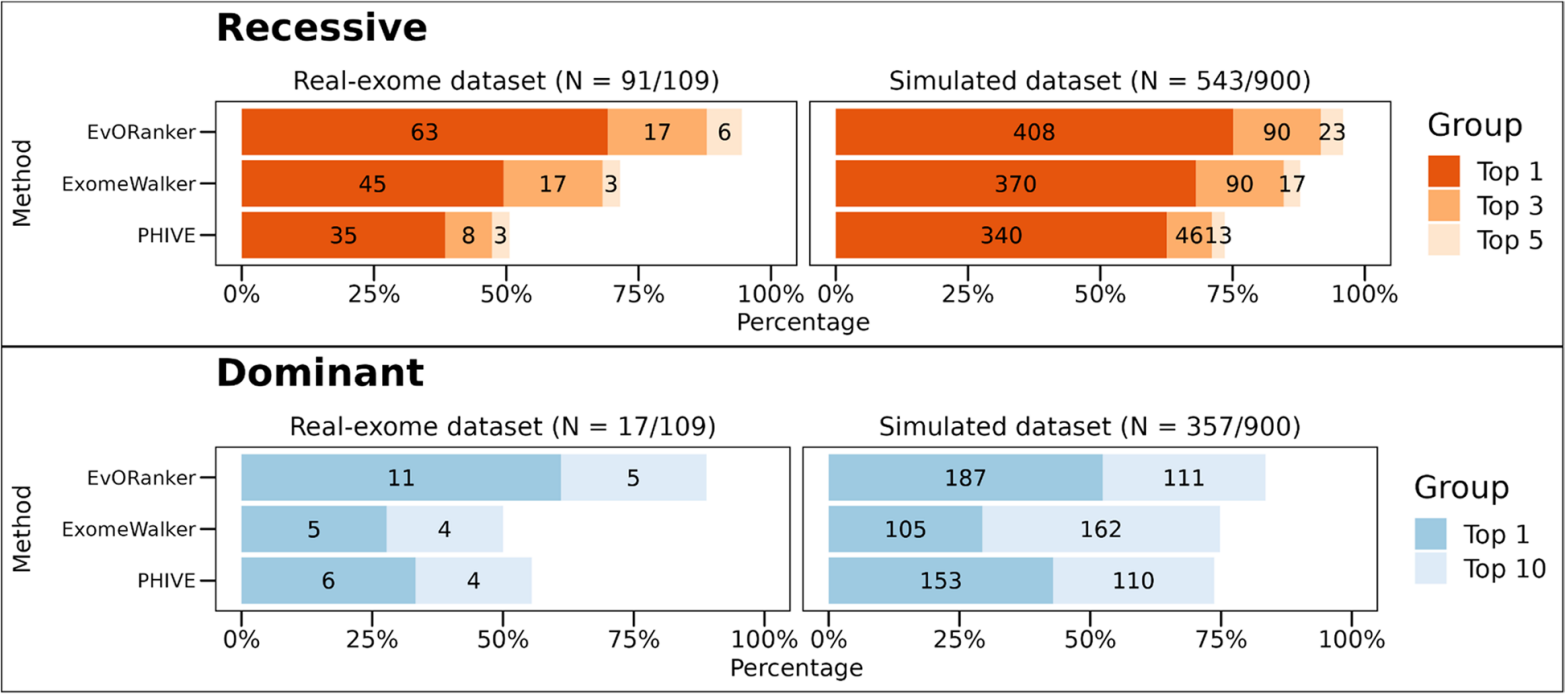
EvORanker outperforms two other algorithms (ExomeWalker and PHIVE). The performance of each algorithm in the 108-exome dataset and the simulated dataset (shuffled three times) was measured by examining the ranking of the “true” disease-causing gene relative to the other patient genes. The upper bar plot shows results for the autosomal and X-linked recessive cases for the real-exome dataset (left) and the simulated dataset (right). The simulated dataset contains 181 unique recessive cases and 119 unique dominant cases. The results present a compilation of three separate independent shuffles totaling 900 simulations. The lower bar plot shows results for the autosomal and X-linked dominant cases. The *y*-axis indicates the tested algorithms, and the *x*-axis indicates the percentage of cases where the “true” disease gene was ranked at the top or within the top 5 genes relative to the other candidate genes in recessive cases. In dominant cases, the percentage indicates whether the “true” gene was ranked at the top or within the top 10 genes. EvORanker outperformed ExomeWalker and PHIVE in both recessive and dominant diseases in both datasets

Using the 109-exome and simulated datasets, we compared the performance of EvORanker to the gene-prioritization stage of ExomeWalker [8] and PHIVE [3]. ExomeWalker prioritizes genes based on protein-protein interaction, while PHIVE uses mouse phenotypic data. To ensure a fair comparison, we chose to compare to ExomeWalker [8] and PHIVE [3] because both adopt a similar strategy to EvORanker. Unlike Phenolyzer [59], these methods do not rely on pre-existing knowledge about known disease genes. The comparison was performed using the exome and simulated datasets with the same input HPO terms. However, since ExomeWalker and PHIVE are not well-suited for CNV analysis, we omitted from this analysis one exome where the causative variant was a large deletion encompassing the *NPRL3* gene. The results of the 108-exome dataset showed that EvORanker outperformed either one or both ExomeWalker and PHIVE in 74% (80/108) of the cases and outperformed both tools in 30% (32/108) of the cases (Fig. 9, Additional file 2: Fig. S10). On the other hand, either one or both of the other tools outperformed EvORanker in 20% (22/108) of the cases (Additional file 2: Fig. S10). For the simulated dataset, EvORanker outperformed both ExomeWalker and PHIVE (Fig. 9).

### Solving the unsolved: candidate genes in reanalysis of patient exomes

We then initiated the application of EvORanker to identify novel disease-causing candidate genes in families with negative clinical exome results. To illustrate its effectiveness, we present two cases where we successfully resolved previously unsolved exomes.

#### Family 1

**Fig. 10:**
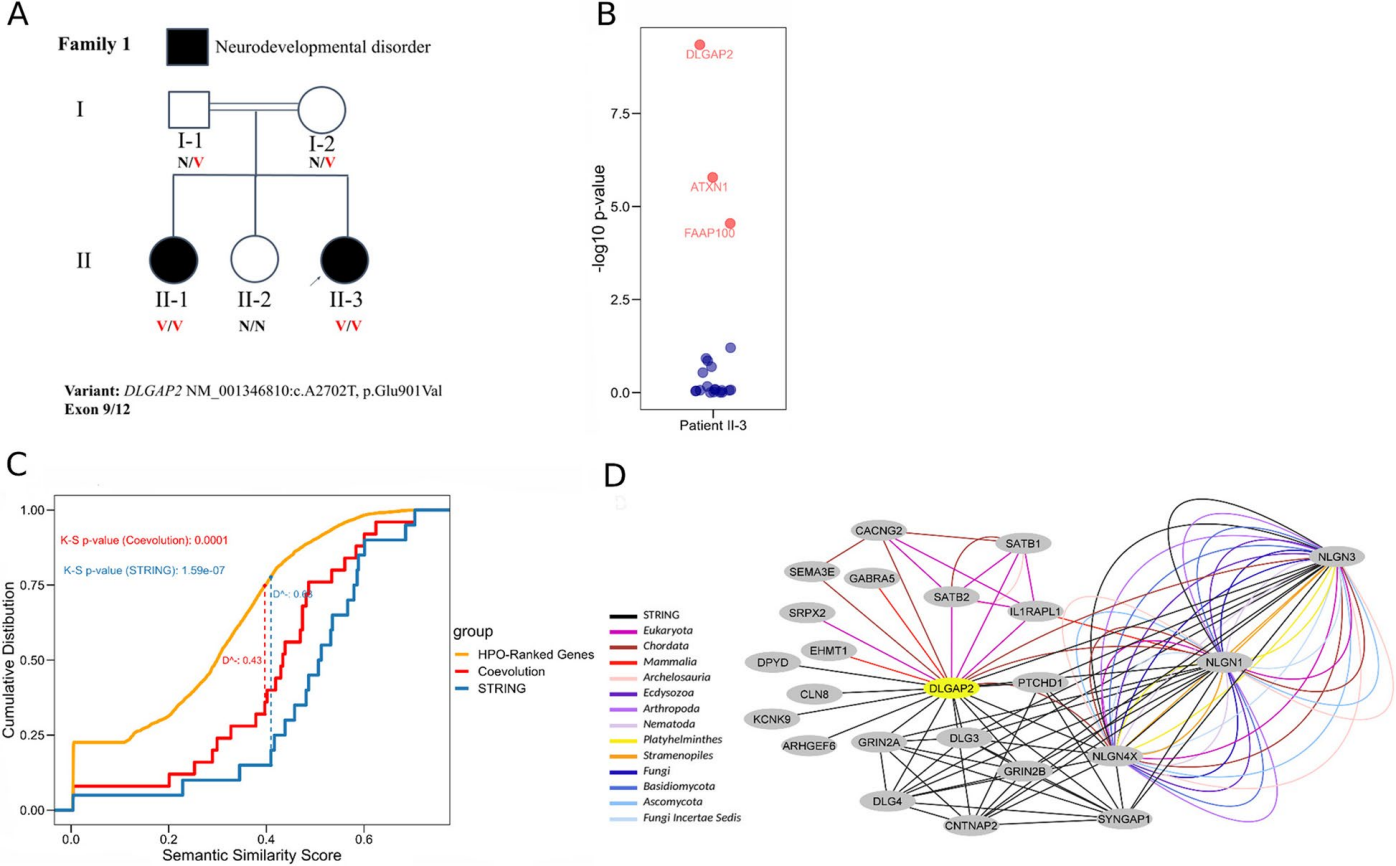
EvORanker identifies *DLGAP2* as a novel gene underlying a neurodevelopmental phenotype. **A** Pedigree: In a consanguineous family affected children have psychomotor delay and dysphasia, hyperactivity, and poor attention span. Shown is the segregation of the *DLGAP2* NM_001346810:c.A2702T, p.Glu901Val variant. N, normal allele; V, variant allele. **B** EvORanker results: *DLGAP2* is ranked as the top candidate relative to the other patient candidates. The *x*-axis indicates the proband (patient II-3), and the *y*-axis indicates the EvORanker -log(10) *p*-value obtained from running the K-S test using the co-evolved and STRING-interacting genes with each patient gene. Red dots indicate significant *p*-values, and dark blue dots indicate non-significant *p*-values. *DLGAP2* was the only gene that co-segregated with the phenotype in family 1. **C** One-sided, two-sample Kolmogorov–Smirnov model. The *x*-axis indicates the semantic similarity score obtained by the *OntologySimilarity* tool in relation to the patient’s (II-3, family 1) phenotypes (HP:0001263, HP:0002357, HP:0000752, HP:0000736). The *y*-axis indicates the cumulative distribution. The orange line corresponds to the empirical distribution of all genes listed in the HPO database, ranked according to semantic similarity. The red line represents the empirical distribution of the genes coevolved with *DLGAP2*, and the blue line represents the empirical distribution of the genes interacting with *DLGAP2* based on STRING. The red dashed line indicates the D statistic representing the maximum vertical distance between the empirical cumulative distribution functions of the HPO-ranked genes and the genes coevolved with *DLGAP2*. The blue dashed line indicates the D statistic measured by the distance between the empirical cumulative distribution functions of the HPO-ranked genes and the genes interacting with *DLGAP2* based on STRING. Both coevolution and STRING-based analysis yielded significant *p*-values corresponding to the D statistic. **D** Coevolution and STRING-based subnetwork showing the patient’s phenotype-related genes coevolving with the *DLGAP2* gene. The dark grey node in the network indicates *DLGAP2* and the light grey nodes represent the phenotype-related genes. The black edges represent STRING interactions, and the colored edges represent the clade where two genes co-evolve. The network exhibits a group of phenotype-related correlated genes that have not been identified by the STRING database (*EHMT1*, *IL1RAPL1*, *SATB2*, *GABRA5*, *SRPX2*, *SEMA3E*, *CACNG2*)

We utilized EvORanker to analyze the exome data of a patient with an undiagnosed neurodevelopmental disorder for which no disease-causing variant was identified. The patient and one of her siblings displayed symptoms of global psychomotor delay, dysphasia, and attention-deficit hyperactivity disorder (ADHD) (Fig. 10A). By employing the previously described analysis steps and inputting the HPO terms HP:0001263, HP:0002357, HP:0000752, and HP:0000736, EvORanker prioritized *DLGAP2* as the top candidate gene (Fig. 10B). Further analysis revealed a strong correlation between *DLGAP2* and several genes related to similar phenotypes to that of the patient, such as *GRIN2A*, *NLGN1*, *CNTNAP2*, *SRPX2*, *SYNGAP1*, *GABRA5*, *DLG3*, *SATB1*, *PTCHD1*, *ARHGEF6*, and *NLGN4X* (Fig. 10C, D, Additional file 2: Fig. S11). These “phenotype-related” genes were significantly enriched within the top co-evolving and STRING-interacting genes with *DLGAP2* (combined Fisher *p*-value = 1.65 × 10^−6^) (Fig. 10C, Additional file 2: Fig. S11). Additionally, *DLGAP2* was ranked as the top gene by both PHIVE [3] and ExomeWalker [8] but ranked 10th by Phenolyzer tool [59].

The high ranking of *DLGAP2* by EvORanker prompted us to further research the *DLGAP2* variant. The *DLGAP2* variant (NM_001346810:c.A2702T, p.Glu901Val) is strongly conserved and not found in the gnomAD population frequency database [42] nor in our in-house database. Both affected siblings were homozygous for the variant, and it was the only variant that co-segregated with the phenotype in the family (Fig. 10A). The variant is positioned on the third nucleotide preceding the splice donor site within exon 9 (out of 12 exons) of the *DLGAP2* gene. It is predicted to alter gene splicing by different prediction tools (e.g., SpliceAI [44]). Since the *DLGAP2* gene is minimally expressed in whole blood, a minigene splicing assay was performed to assess the effect of the c.A2702T variant on gene splicing (Additional file 2: Fig. S12). The minigene assay results showed that the variant led to the activation of a cryptic splice site and aberrant splicing (Additional file 2: Fig. S12). Sequencing of the RT-PCR product of the mutant construct showed a 4-bp deletion (GAAA del) (Chr8:1,626,792–1,626,795), resulting in a frameshift and premature termination after 59 codons (Additional file 2: Fig. S12).

#### Family 2

**Fig. 11:**
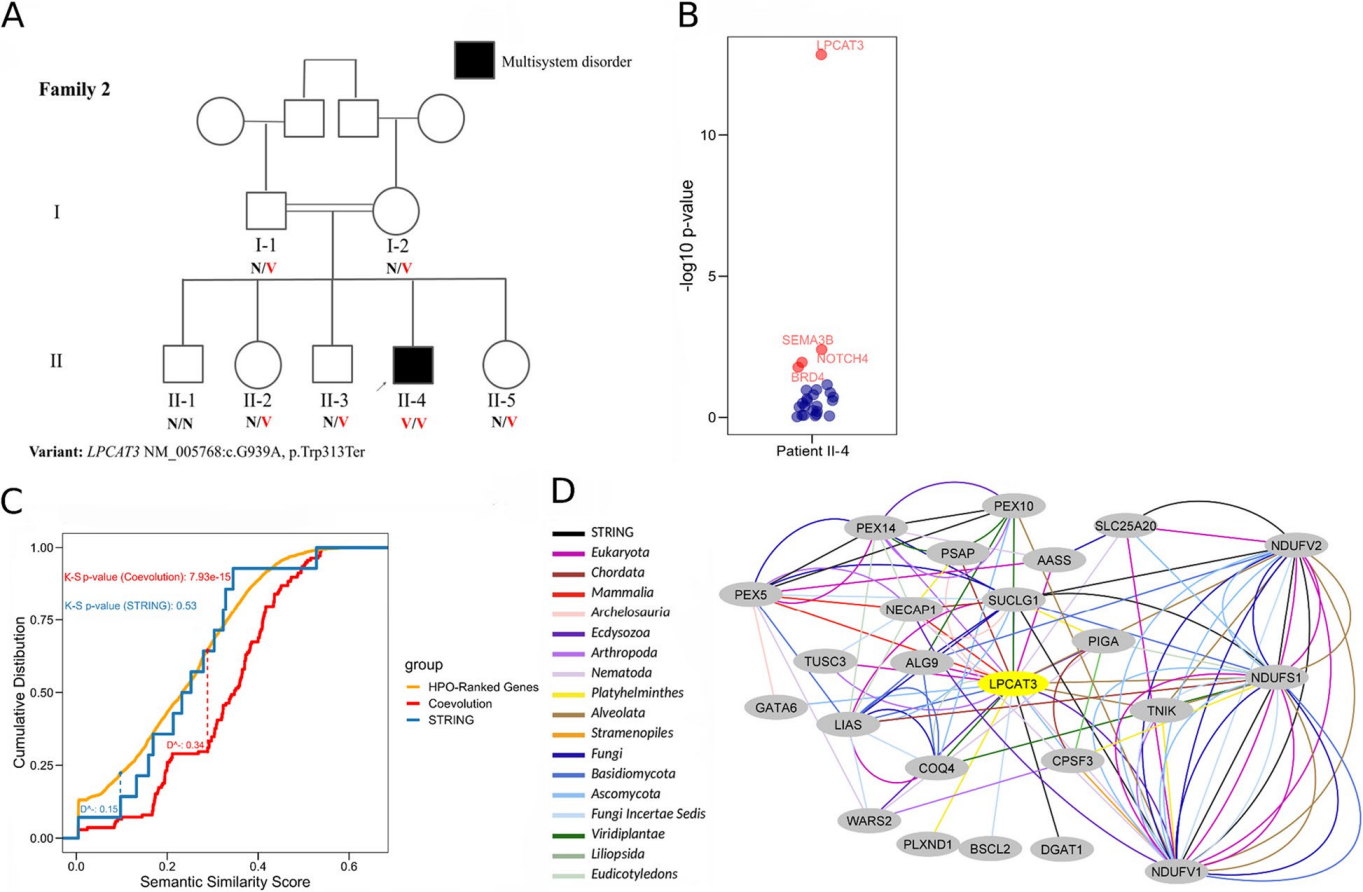
EvORanker identifies *LPCAT3* as a novel gene underlying a multisystem disorder. **A** Pedigree of a consanguineous family. The affected son has failure to thrive, chronic diarrhea with recurrent abdominal pain, muscle atrophy, elevated liver enzymes, and high creatine kinase levels. Shown is the segregation of the *LPCAT3* NM_005768:c.G939A, p.Trp313Ter variant. N, normal allele; V, variant allele. **B** EvORanker results: *LPCAT3* is ranked as the top candidate relative to other candidate genes. The *x*-axis indicates the proband (patient II-4), and the *y*-axis indicates the combined -log10 *p*-value obtained from running the K-S test using the co-evolved and STRING-interacting genes with each patient gene. Red dots indicate significant *p*-values, and dark blue dots indicate non-significant *p*-values. *LPCAT3* was the only gene that co-segregated with the phenotype in family 2. **C** One-sided, two-sample Kolmogorov–Smirnov model. The *x*-axis indicates the semantic similarity score obtained by the OntologySimilarity tool in relation to the patient’s (II-4, family 2) phenotypes (HP:0001508, HP:0002910, HP:0002574, HP:0002028, HP:0003236, HP:0003202). The *y*-axis indicates the cumulative distribution. The orange line corresponds to the empirical distribution of all genes listed in the HPO database, ranked according to semantic similarity. The red line indicates the empirical distribution of the genes coevolved with *LPCAT3*, and the blue line indicates the empirical distribution of the genes interacting with *LPCAT3* based on STRING. The red dashed line indicates the D statistic representing the maximum vertical distance between the empirical cumulative distribution functions of the HPO-ranked genes and the genes coevolved with *LPCAT3*. The blue dashed line indicates the D statistic measured by the distance between the empirical cumulative distribution functions of the HPO-ranked genes and the genes interacting with *LPCAT3* based on STRING. Only coevolution-based analysis yielded significant *p*-values corresponding to the D statistic. **D** Coevolution and STRING-based subnetwork showing the patient’s phenotype-related genes coevolving with the *LPCAT3* gene. The yellow node in the network indicates *LPCAT3* and the light grey nodes represent the phenotype-related genes. The black edges represent STRING interactions, and the colored edges represent the clade where two genes co-evolve. We demonstrate that our clade-wise NPP approach uncovered correlations between *LPCAT3* and *phenotype-related genes* that were not captured by STRING

We applied EvORanker to the exome data of a patient diagnosed with a multisystem disease including failure to thrive, recurring abdominal pain, chronic diarrhea, skeletal muscle wasting, elevated liver enzymes, and high levels of creatine kinase (Fig. 11A). The patient is of consanguineous parentage and is the sole affected individual in the family (Fig. 11A). Using HPO terms corresponding to the patient’s phenotype (HP:0001508, HP:0002910, HP:0002574, HP:0002028, HP:0003236, HP:0003202), EvORanker prioritized *LPCAT3* as the top patient candidate gene (Fig. 11B). *LPCAT3* demonstrated strong coevolution signals with genes related to the patient’s phenotype (*PYGL*, *DLD*, *TXNRD2*, *COG8*, *SUCLG1*, *MVK*, *SMAD4*, *CPT1A*) in the plant (*Viridiplantae* and *Eudicotyledons*), *Mammalia*, and *Fungi* kingdoms (Fig. 11C, D, Additional file 2: Figs. S13, S14 and S15). The genes that showed the strongest coevolution with *LPCAT3* were significantly enriched within the *phenotype-related genes* (*p*-value = 7.93 × 10^−15^) (Fig. 10C, Additional file 2: Fig. S13). Conversely, the genes that interacted with *LPCAT3* through STRING did not exhibit significant enrichment within the *phenotype-related genes* (*p*-value = 0.53) (Fig. 10C, Additional file 2: Fig. S13). Despite this, *LPCAT3* still had the most significant *p*-value among all candidates based on the combined EvORanker score (Fisher combined *p*-value = 1.42 × 10^−13^). *LPCAT3* was ranked third by ExomeWalker [8], excluded by PHIVE [3] and ranked 8th by Phenolyzer [59]. The proband (II-4) was homozygous for a truncating variant in exon 9 of the *LPCAT3* gene (NM_005768:c.G939A, p.W313X) (Fig. 11A). This variant was not found in the gnomAD population frequency database [42] nor in our in-house database. The *LPCAT3* variant was the only variant among the *patient candidate genes* that co-segregated with the phenotype in the family (Fig. 11A).

Complete knockout of *LPCAT3* in mice results in premature death. However, tissue-specific knockouts in the liver and intestines have been documented, with the latter causing impaired growth and abnormal enterocyte morphology along with enterocyte lipid accumulation (Rong et al., 2015). Liver-specific knockouts in mice display a decrease in plasma triglycerides and an occurrence of hepatosteatosis (Rong et al., 2015). The patient from family 2 demonstrated anomalies in both the intestine and liver. Duodenal biopsies showed nodular lesions in the duodenal bulb and the descending portion of the duodenum with atrophic mucosa suggestive of severe enteropathy. Fragments of duodenal mucosa showed partial villous blunting with a mild increase of lamina propria lymphoplasmacytic cell infiltrate. Liver enzymes revealed a reduced ratio of aspartate aminotransferase (AST)/alanine aminotransferase (ALT) ratio, suggesting fatty liver disease, along with reduced plasma triglycerides (34 mg/dL) and HDL levels (27.1 mg/dL). These findings suggest *LPCAT3* as a potential causative gene for the disease in the proband of this family.

### EvORanker web tool

The EvORanker web tool (https://ccanavati.shinyapps.io/EvORanker/) is an easy-to-use and user-friendly decision support tool built for geneticists and researchers in the NGS field (Additional file 2: Fig. S16). The user submits a set of HPO terms describing the patient’s medical condition and the patient’s candidate genes, preferably genes that survived variant filtering. The algorithm then performs the aforementioned analyses and returns the outputs in two stages:

1. Step 1: If the queried gene is already listed in the HPO database, a semantic similarity score (ranging from 0 to 1) reflecting the similarity of the gene’s associated HPO terms to the user’s input HPO terms is calculated using the OntologySimilarity package [51] and is indicated in a table output in the “Step 1: Semantic Similarity-based Prioritization” tab.
2. Step 2: In the case where none of the queried genes are listed in the HPO dataset or where none had a high or sufficient semantic similarity score (i.e., a non-diagnostic case), the user can navigate to co-evolution and STRING-based gene prioritization. The output is a table containing each queried gene and the corresponding EvORanker *p*-value. The EvORanker *p*-value is the result of Fisher’s combined test obtained by integration of multi-clade phylogenetic profiling and STRING-based analysis as described above.

EvORanker also provides useful visualizations of the results, including a bar plot of the ranked genes by EvORanker, and a co-evolution and STRING subnetwork generated upon click of any queried gene in the “Step 2” results table. The network highlights the HPO-related genes co-evolving with the query gene in addition to edges representing STRING interactions. Additionally, the user can retrieve more detailed co-evolutionary information including the clade where every two genes co-evolve, the co-evolutionary rank of the HPO-related genes with each query gene, and can inspect gene enrichment results of the coevolving genes and STRING-interacting genes with each query gene. The web interface is available at the following link: https://ccanavati.shinyapps.io/EvORanker/. Recognizing the need to analyze a larger number of genes than recommended for the web tool due to memory constraints, we have established a GitHub repository (https://github.com/ccanavati/EvoRanker) [65]. This repository allows users to access the tool and input an expanded number of genes, accommodating their requirements.

## Discussion

Clinical elucidation of genetic variants in connection to a patient’s phenotype is a time-consuming and costly element in the genomic diagnosis of rare genetic diseases. To address this issue, several computational algorithms have been developed over the years to prioritize candidate genes based on the patient’s phenotype using different sources of information, such as protein-protein interactions, data mining, and gene expression [3, 8–10, 12–14]. Nevertheless, although PP was successfully used to identify novel disease genes [25, 26, 29, 30, 66], we are not aware of any tool that systematically utilizes clade-based phylogenetic profiling to prioritize patient candidate genes. Herein, we described EvORanker, an algorithm that employs multi-scale phylogenetic profiling and gene interaction data from the STRING database [33] to analyze “unsolved” WES/WGS cases in search of novel genetic causes of disease. This algorithm integrates unbiased comparative genomic analysis with publicly available gene data, including function and interactions.

Multi-scale phylogenetic profiling is particularly valuable for identifying disease associations for poorly annotated genes. The ability to conduct analysis of every gene independently of existing knowledge expands the scope of disease-gene discovery. This is particularly important in light of the “rich get richer” phenomenon, where genes that have already been studied receive disproportionate attention, while poorly annotated genes are often overlooked. Among the 6260 tested knockout genes that exhibit a phenotype in mice and have an ortholog in humans, EvORanker was able to link 41% of these genes to the disease phenotype observed in mice (Fig. 8, Additional file 2: S9). This highlights the potential of EvORanker to discover new disease genes and expand our understanding of disease mechanisms.

Furthermore, our study demonstrates the power of our multi-clade concept in capturing co-evolution, as shown by our ability to more effectively identify the “true” disease-causing genes across multiple clades, beyond just Eukaryota or Animalia clades (Figs. 3 and 4, Additional file 2: Fig. S5). This is aligned with the notion that multi-clade phylogenetic profiling-based methods more effectively capture co-evolution [29, 30]. Importantly, our clade-wise NPP approach revealed correlations between genes that could not be anticipated using other omics (Fig. 11C, D, Additional file 2: Fig. S13). The integration of NPP with STRING leads to increased efficiency of EvORanker (Fig. 5), especially for newly annotated genes, and highlights the complementarity of these two datasets. In future studies, we may contemplate incorporating additional datasets into the algorithm, such as mouse and zebrafish knockout data, and other sources for protein-protein interaction networks, by utilizing similar concepts.

We benchmarked our tool using both real patient exome data, in addition to simulated data. The utilization of actual patient data enhances the translational potential of our findings and underscores the clinical relevance of our tool. EvORanker ranked the “true” gene within the top 5 in 95% of the patient-exome dataset. On the other hand, failed to rank the “true” gene within the top 5 for recessive diseases and within the top 10 for dominant diseases in 6/109 exomes. Further investigation revealed that in 3 of those cases (*TBL1XR1*, *NHLRC2*, *ADGRG1*), the HPO terms used as input into the algorithm were both insufficient and non-specific (Additional file 1: Table S1). This highlights the importance of precise selection of HPO terms to achieve accurate results. Notably, our results remained consistent across both the real-patient and simulation datasets, further validating the practical utility and effectiveness of our tool in real-world applications.

We applied EvORanker on two unresolved exomes in which previous clinical whole exome sequencing (WES) did not identify a known genetic cause. In the first case (family 1), the *DLGAP2* gene was ranked as the top candidate for a proband with a neurodevelopmental disorder (Fig. 10B). *DLGAP2* plays a role in the molecular organization of neuronal synapses and neuronal cell signaling [67]. The pathogenicity of the NM_001346810:c.A2702T, p.Glu901Val variant observed in *DLGAP2* was validated by demonstrating its effect on splicing (Additional file 2: Fig. S12). Homozygous knockout mice for *DLGAP2* exhibit novelty-induced hyperactivity, increased aggression, impaired reverse learning, decreased dendritic spine density, and synaptopathy [68] providing further support for the association of *DLGAP2* with the patient’s phenotype. Furthermore, *DLGAP2* was hypothesized to be a strong candidate for neurodevelopmental and behavioral phenotypes observed in patients harboring 8p23.2-pter microdeletions including *DLGAP2* and four other genes [69]. Notably, our analysis using NPP revealed a group of *DLGAP2*-associated genes not detected by STRING (Fig. 10D), providing new avenues for investigating the role of *DLGAP2* in the nervous system.

In the second “unsolved” exome, only NPP ranked the *LPCAT3* gene as the top candidate (family 2, Fig. 11C, Additional file 2: Fig. S13). This ranking of *LPCAT3* was achieved by the detection of novel functional associations with *phenotype-related genes* based on co-evolution (*PYGL*, *DLD*, *TXNRD2*, *COG8*, *SUCLG1*, *MVK*, *SMAD4*, *CPT1A*) (Fig. 11D). These *phenotype-related genes* showed significant coevolution with *LPCAT3* in the clades of *Viridiplantae*, *Eudicotyledons*, *Mammalia*, and *Fungi* (Fig. 11D, Additional file 2: Figs. S11 and S14), pointing towards novel associations not captured by STRING [33]. These findings are supported by phenotypic similarities between the patient and liver and intestinal knockout mice [70], including failure to thrive, enteropathy, and low levels of triglycerides and high-density lipoprotein. *LPCAT3* nullizygous mice exhibit postnatal death [70], making it difficult to study the global effects of *LPCAT3* knockdown. Although a recent report linked *LPCAT3* overexpression to skeletal muscle myopathy [71], further research is needed to understand the role and mechanism of *LPCAT3* in this condition. Taken together, these results underscore the potential of clade-based NPP to predict functional associations with phenotype-relevant genes. Further validation in additional patients with variants in these genes is warranted to confirm their roles as novel disease-associated genes. Subsequent functional validation studies are crucial to better understand the mechanisms of disease pathogenesis.

EvORanker is entirely gene-based, making it adaptable to various sequencing experiments and accessible for users with minimal computational knowledge. In addition to providing a ranked gene list, EvORanker offers the ability to explore evolutionary and STRING-based gene networks across multiple clades. A recommended strategy for users is to first examine the ranking of genes based on the OntologySimilarity semantic similarity score [51], in the event that one of the candidate genes is already listed in the HPO database. If not, the user can then evaluate the ranking of genes based on the EvORanker score, where a novel association between the gene and input phenotypes may be discovered. The EvORanker server is freely available at https://ccanavati.shinyapps.io/EvORanker/, which will be updated on a regular basis. We also created a GitHub repository (https://github.com/ccanavati/EvoRanker) [65] which allows users to access the tool and input an expanded number of genes.

## Conclusions

In summary, our work introduces EvORanker as a powerful tool in the genomic diagnostic landscape. By integrating multi-scale phylogenetic profiling and STRING-based gene interaction data, EvORanker offers a unique and effective approach to prioritize candidate genes in “unsolved” cases identified through whole-exome and whole-genome sequencing. Our validation using real patient exome data and simulation data demonstrates EvORanker’s robust capability to consistently prioritize the “true” gene, showcasing its reliability and translational potential in research applications.

The effectiveness of EvORanker in identifying candidate disease genes, as demonstrated by the identification of *DLGAP2* and *LPCAT3* in previously unresolved cases, highlights its potential to contribute to our understanding of disease mechanisms. Moreover, its adaptability, user-friendly interface, and accessibility without extensive computational expertise make EvORanker a valuable asset for researchers. As we navigate the intricate landscape of rare genetic diseases, EvORanker stands as a promising tool, offering not only a ranked gene list but also insights into STRING-based and evolutionary gene networks across multiple clades. We believe that the adoption of EvORanker will contribute significantly to advancing genomic diagnostics in the pursuit of unraveling the genetic mysteries underlying rare diseases.

### Supplementary Information

**Additional file 1: Table S1.** Clinical and Genetic Characteristics of the 109-Patient Exome Dataset. **Table S2.** ClinVar-Simulated Genetic Variants and Associated Phenotypes. **Table S3.** Newly added disease-gene entries in the Human Phenotype Ontology (HPO) database.

**Additional file 2: Figure S1.** Parameter combinations and EvORanker performance. **Figure S2.** The 16 clades used in the phylogenetic profiling-based algorithm. **Figure S3.** Cutoff values and EvORanker performance. **Figure S4.** Distribution of the number of* patient candidate genes* that passed the variant filtering criteria in autosomal and x-linked recessive (red) and dominant (dark blue) cases in the (A) patient exome dataset and the (B) simulated dataset (shuffled three times). **Figure S5.** Contribution of each of the 16 clades to the overall performance of the EvORanker. **Figure S6.** Radar plot showing the ranking of the “true” disease-causing gene (top 1, top 10, or NULL) using EvORanker (red), NPP (golden), and STRING (dark blue). **Figure S7.** Evaluating EvORanker Performance across three independent spike shuffles. **Figure S8.** Performance of NPP versus STRING using the 109-patient exome dataset across the years. **Figure S9.** Comparison of EvORanker and Phenolyzer in identifying true disease gene candidates. **Figure S10.** Radar plot showing the ranking of the “true” disease-causing gene (top 1, top 10, or NULL) using EvORanker (red), PHIVE (golden), and ExomeWalker (blue). **Figure S11.** Distributions of the *HPO-ranked genes*, the co-evolved genes, and STRING-interacting genes with *DLGAP2*. **Figure S12.** Effect of *DLGAP2* p.E901V on splicing. **Figure S13.** Density distributions of the *HPO-ranked genes*, the co-evolved genes, and STRING-interacting genes with *LPCAT3*. **Figure S14.** Clades differentially predict the functional interaction between the *phenotype-related genes* and *LPCAT3*. **Figure S15.** The Phylogenetic profiles of *LPCAT3* and patient HPO-related genes across 1,028 eukaryotes. **Figure S16.** Homepage of the EvoRanker web interface.

## Data Availability

All the algorithm-related data files on which the conclusions of the paper rely on are publicly available on GitHub at the following link: https://github.com/ccanavati/EvoRanker [65]. The dataset comprising the exome sequencing data of 109 patients at Istishari Arab Hospital in Ramallah, Palestine, is available through Professor Moien Kanaan. It was employed for benchmarking purposes in the current study. However, due to licensing constraints, these data are not publicly accessible. Researchers interested in accessing the dataset may do so upon making a reasonable request. To initiate the request, individuals are required to contact Professor Moien Kanaan directly via email (moien.kanaan@iah.ps). Please note that the process of obtaining access involves a meeting with professor Moien Kanaan, during which the interested party will be required to provide details on how the data will be utilized, secured, and maintained to ensure the privacy and confidentiality of the patients. This may include a discussion on data security measures, protocols for restricting access to authorized personnel, and assurances that the data will not be made available to third parties. The timeline for granting access will be determined on a case-by-case basis, taking into consideration the nature of the request and the required ethical and legal approvals. The VCF files for the 300 genomes utilized in the simulation analysis were obtained from the 1000 Genome Project [39], accessible at (http://ftp.1000genomes.ebi.ac.uk/vol1/ftp/release/20100804/) [40].
