# Supplementary material for: Using multi-scale genomics to associate poorly annotated genes with rare diseases
Source: Genome Med. 2024 Jan 4;16:4. doi: 10.1186/s13073-023-01276-2 (PMC10765705; doi:10.1186/s13073-023-01276-2)
Supplement: Supplementary file 2 — Additional file 2: Figure S1. Parameter combinations and EvORanker performance. Figure S2. The 16 clades used in the phylogenetic profiling-based algorithm. Figure S3. Cutoff values and EvORanker performance. Figure S4. Distribution of the number of patient candidate genes that passed the variant filtering criteria in autosomal and x-linked recessive (red) and dominant (dark blue) cases in the (A) patient exome dataset and the (B) simulated dataset (shuffled three times). Figure S5. Contribution of each of the 16 clades to the overall performance of the EvORanker. Figure S6. Radar plot showing the ranking of the “true” disease-causing gene (top 1, top 10, or NULL) using EvORanker (red), NPP (golden), and STRING (dark blue). Figure S7. Evaluating EvORanker Performance across three independent spike shuffles. Figure S8. Performance of NPP versus STRING using the 109-patient exome dataset across the years. Figure S9. Comparison of EvORanker and Phenolyzer in identifying true disease gene candidates. Figure S10. Radar plot showing the ranking of the “true” disease-causing gene (top 1, top 10, or NULL) using EvORanker (red), PHIVE (golden), and ExomeWalker (blue). Figure S11. Distributions of the HPO-ranked genes, the co-evolved genes, and STRING-interacting genes with DLGAP2. Figure S12. Effect of DLGAP2 p.E901V on splicing. Figure S13. Density distributions of the HPO-ranked genes, the co-evolved genes, and STRING-interacting genes with LPCAT3. Figure S14. Clades differentially predict the functional interaction between the phenotype-related genes and LPCAT3. Figure S15. The Phylogenetic profiles of LPCAT3 and patient HPO-related genes across 1,028 eukaryotes. Figure S16. Homepage of the EvoRanker web interface. [file 13073_2023_1276_MOESM2_ESM.docx]

# **Supplementary Material**


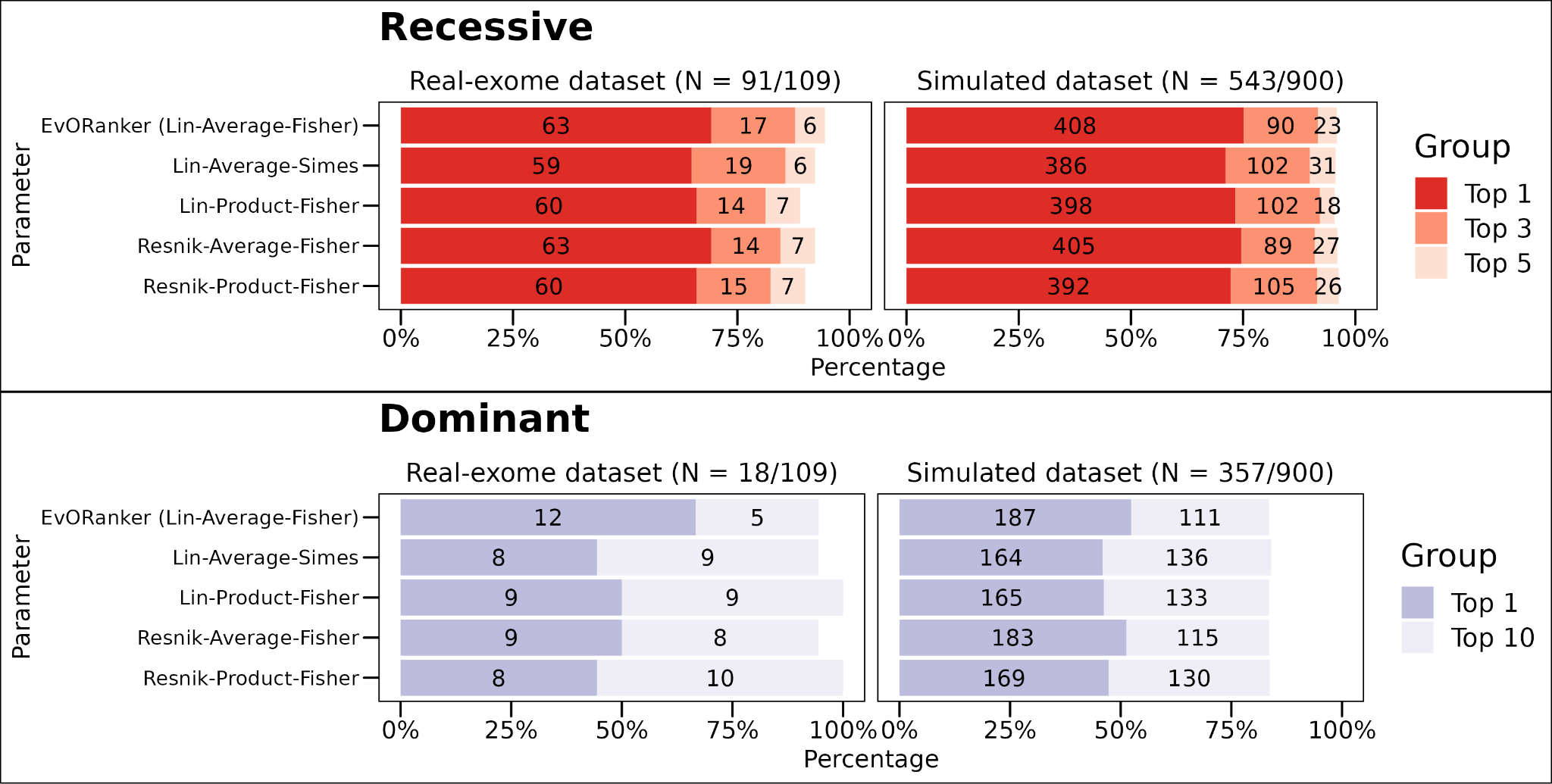


**Figure S1. Parameter combinations and EvORanker performance.** For each patient exome in the 109-patient exome database, and genome in the simulated dataset, different semantic similarity measures (Lin’s, Resnik’s), calculation of similarity between sets of terms (‘product’, ‘best-match-average’) and methods to combine p-values (Fisher’s, Simes’) were tested. The accuracy was measured by examining the ranking of the “true” disease-causing gene relative to the other candidate genes. The upper bar plot shows results for the autosomal and X-linked recessive cases for the real-exome dataset (left) and the simulated dataset (right). The simulated dataset contains 181 unique recessive cases and 119 unique dominant cases. The results present a compilation of three separate independent shuffles totaling 900 simulations. The lower bar plot shows results for the autosomal and X-linked dominant cases. The y-axis indicates the parameter combinations and the x-axis indicates the percentage of cases where the “true” disease gene was ranked at the top, or within the top 3 or top 5 genes relative to the other candidate genes in autosomal recessive cases. In dominant cases, the percentage indicates whether the “true” gene was ranked at the top or within the top 10 genes. Overall, the tested parameter combinations yielded nearly identical results across both datasets. Specifically, combining Lin's method with Fisher's produced slightly improved outcomes. Key: Average - best-match-average.


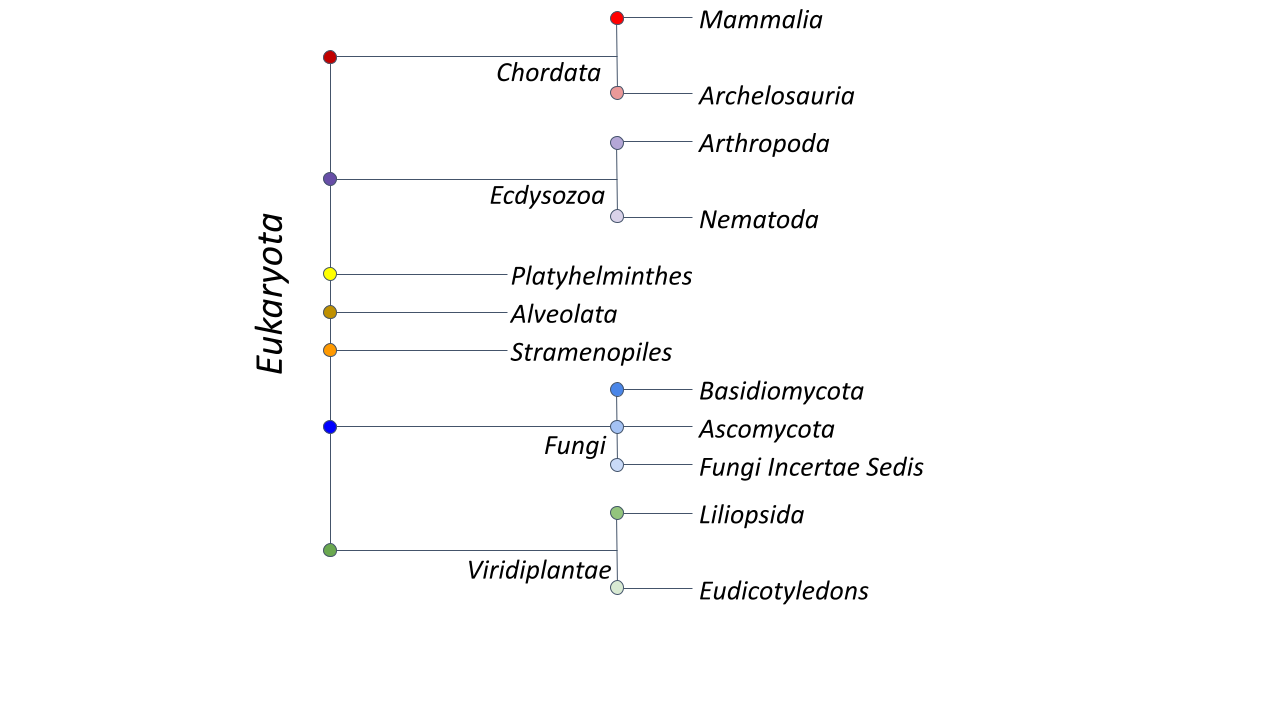


**Figure S2: The 16 clades used in the phylogenetic profiling-based algorithm.** The clades are ordered by hierarchy and distance from humans. Eukaryota encompasses all the clades that branch from left to right. The order of the clades from top to bottom represents the distance from humans (Chordata is the closest clade). The color code used for each clade is applied to all the figures in this paper.


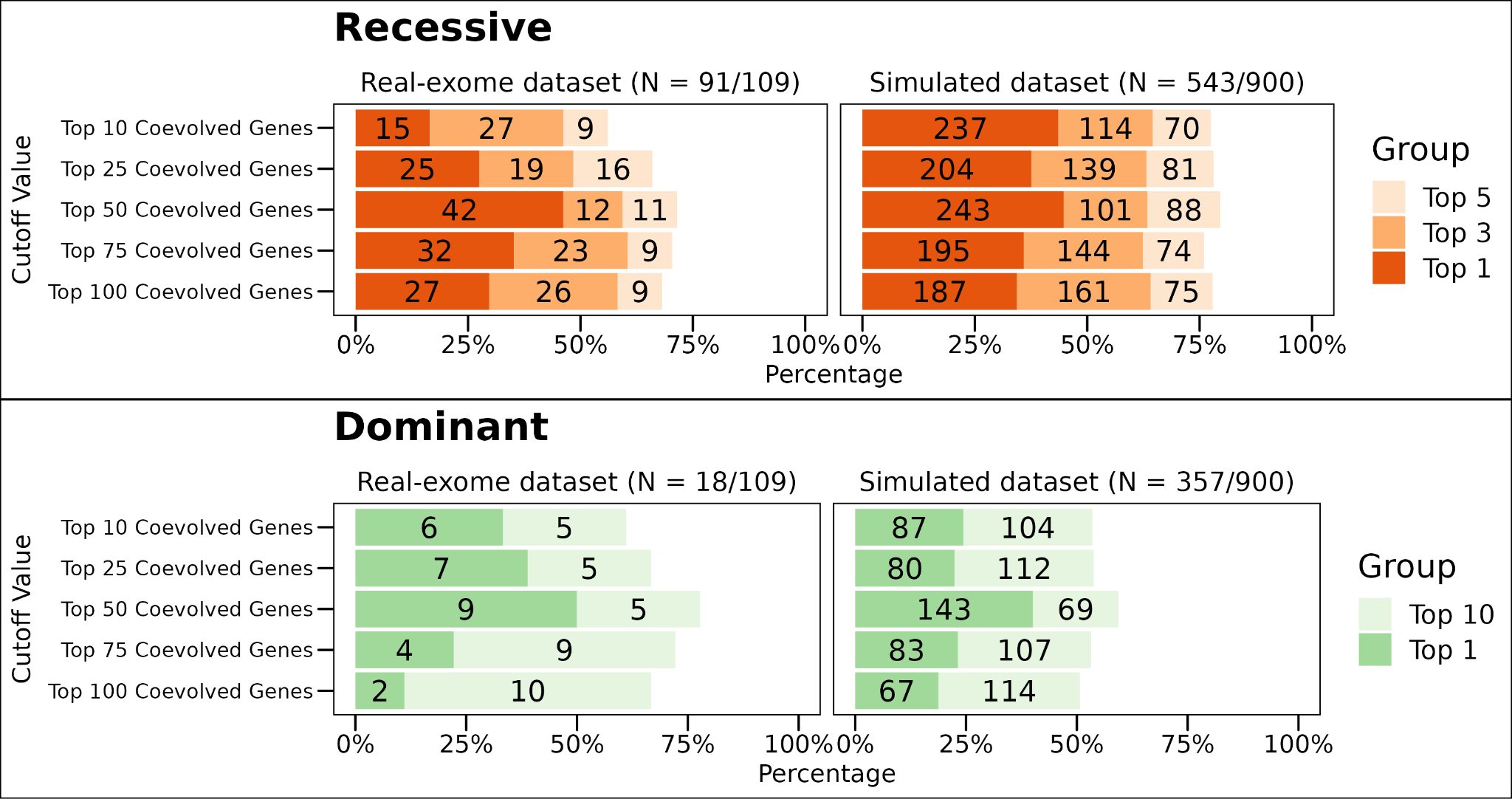


**Figure S3. Cutoff values and EvORanker performance.** For each patient exome in the 109-patient exome database, and genome in the simulated dataset, different cutoff values (top 10, 25, 50, 75, 100) of the co-evolving genes with each *candidate gene* were tested. The accuracy was measured by examining the ranking of the “true” disease-causing gene relative to the other patient genes. The upper bar plot shows results for the autosomal and X-linked recessive cases for the real-exome dataset (left) and the simulated dataset (right). The simulated dataset contains 181 unique recessive cases and 119 unique dominant cases. The results present a compilation of three separate independent shuffles totaling 900 simulations. The lower bar plot shows results for the autosomal and X-linked dominant cases. The y-axis indicates the cutoff values and the x-axis indicates the percentage of cases where the “true” disease gene was ranked at the top, or within the top 3 or top 5 genes relative to the other candidate genes in autosomal recessive cases. In dominant cases, the percentage indicates whether the “true” gene was ranked at the top or within the top 10 genes. Overall, the best performance in ranking the “true” causative gene was achieved by a cutoff value of the top 50 co-evolving genes where the query gene is found to have an ortholog in both datasets.


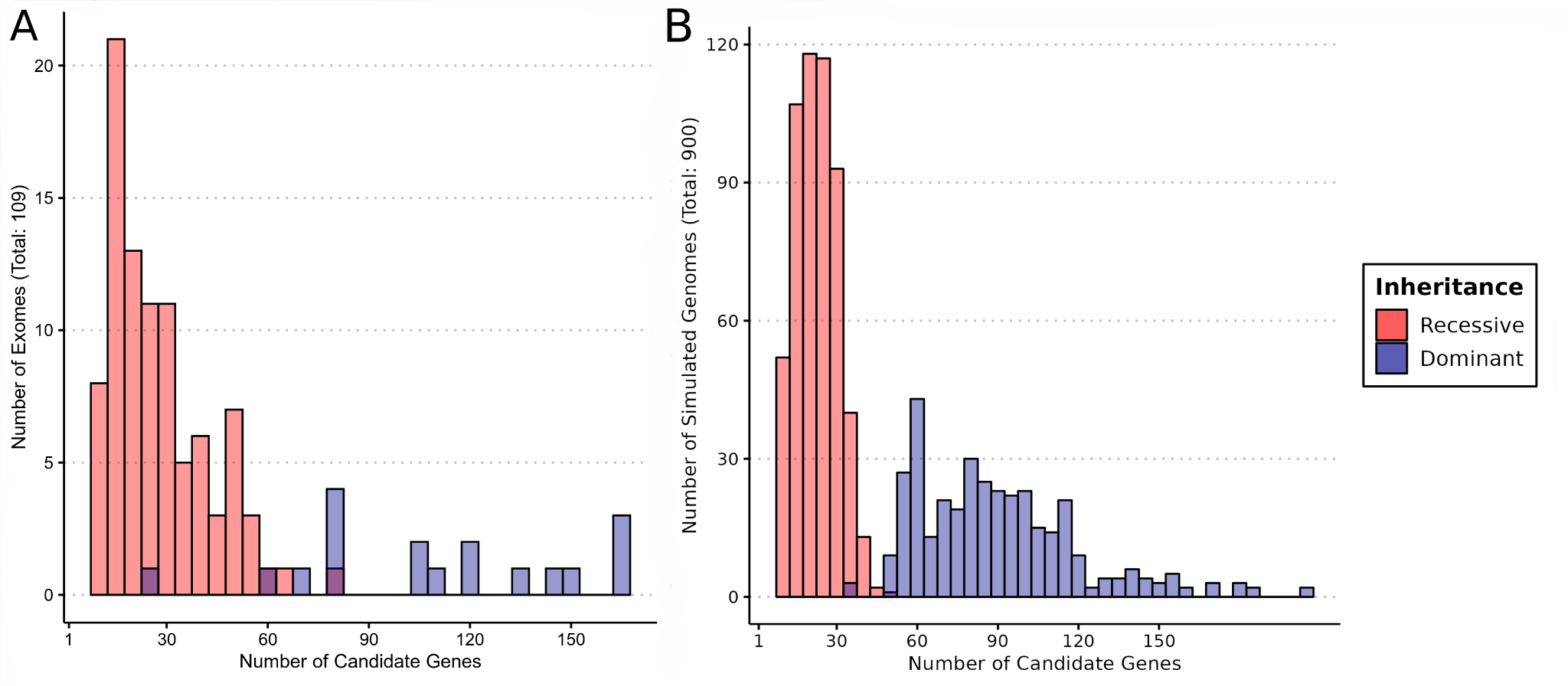


**Figure S4. Distribution of the number of *patient candidate genes* that passed the variant filtering criteria in autosomal and x-linked recessive (red) and dominant (dark blue) cases in the (A) patient exome dataset and the (B) simulated dataset (shuffled three times).** Variant filtering criteria include filtering out the intronic, untranslated region, and ncRNA variants. Nonsense, frameshift, nonsynonymous, and splice‐site variants are prioritized by excluding variants based on their minor allele frequency in gnomAD (https://gnomad.broadinstitute.org), AF_popmax database and Istishari Arab Hospital’s in-house exome database. Variants predicted to be benign by variant effect predictor tools are excluded from the analysis (Table 1).

| 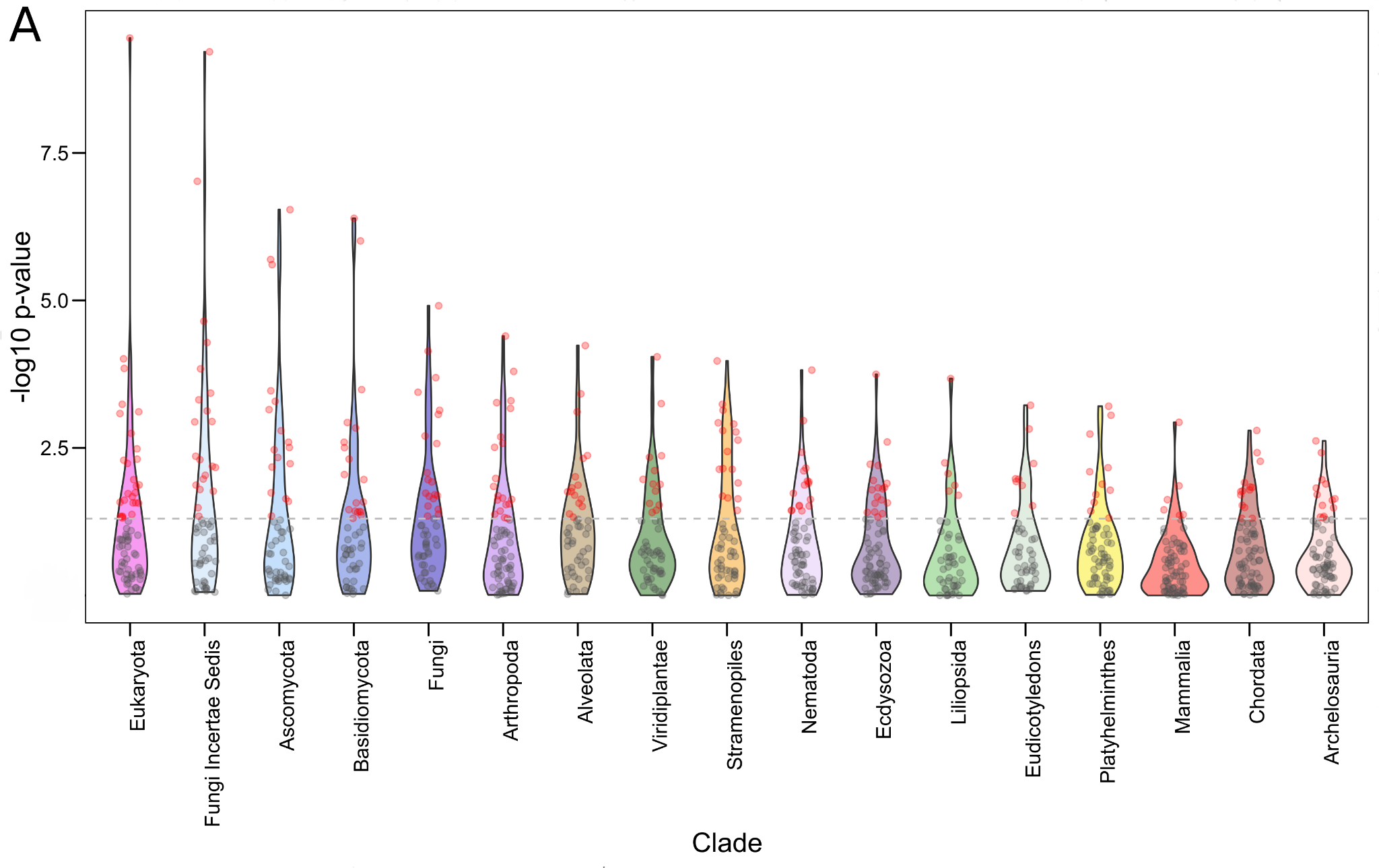 | 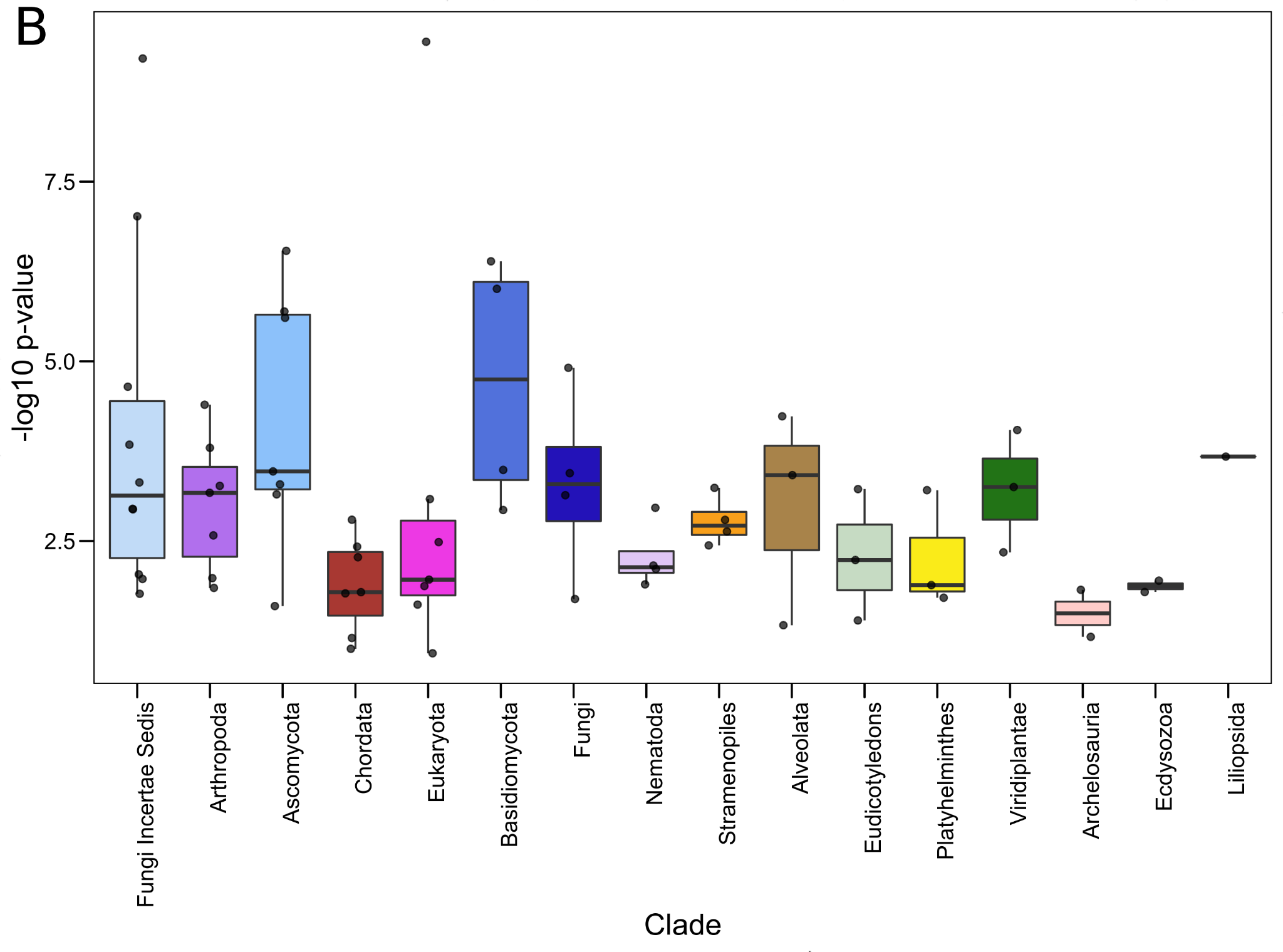 |
| --- | --- |

**Figure S5.** **Contribution of each of the 16 clades to the overall performance of the EvORanker.** This analysis was based on the 71 genes that achieved an overall significant K-S test p-value (<0.05) using the co-evolution analysis. For each of these disease genes, the K-S test was applied using the co-evolving genes with each disease gene within each clade separately. In (A), the x-axis indicates all 16 clades in addition to Eukaryota, and the y-axis indicates the -log10 of the K-S test p-value for each of those genes. The dark grey dots represent non-significant p-values, and the red dots displayed above the dashed horizontal line indicate significant p-values (>-log10(0.05)). (B) For each of the 71 disease genes, the clade that outperformed/ achieved the most significant p-value in comparison to the other clades is displayed. The x-axis indicates the clades and the y-axis represents the -log10 of the K-S test p-value obtained by each clade. *Fungi Incertae Sedis* clade outperformed the other clades in 10/71 (14%) of the cases. On the other hand, *Mammalia* is the only clade that hadn’t outperformed the other clades in any of the genes in this dataset.


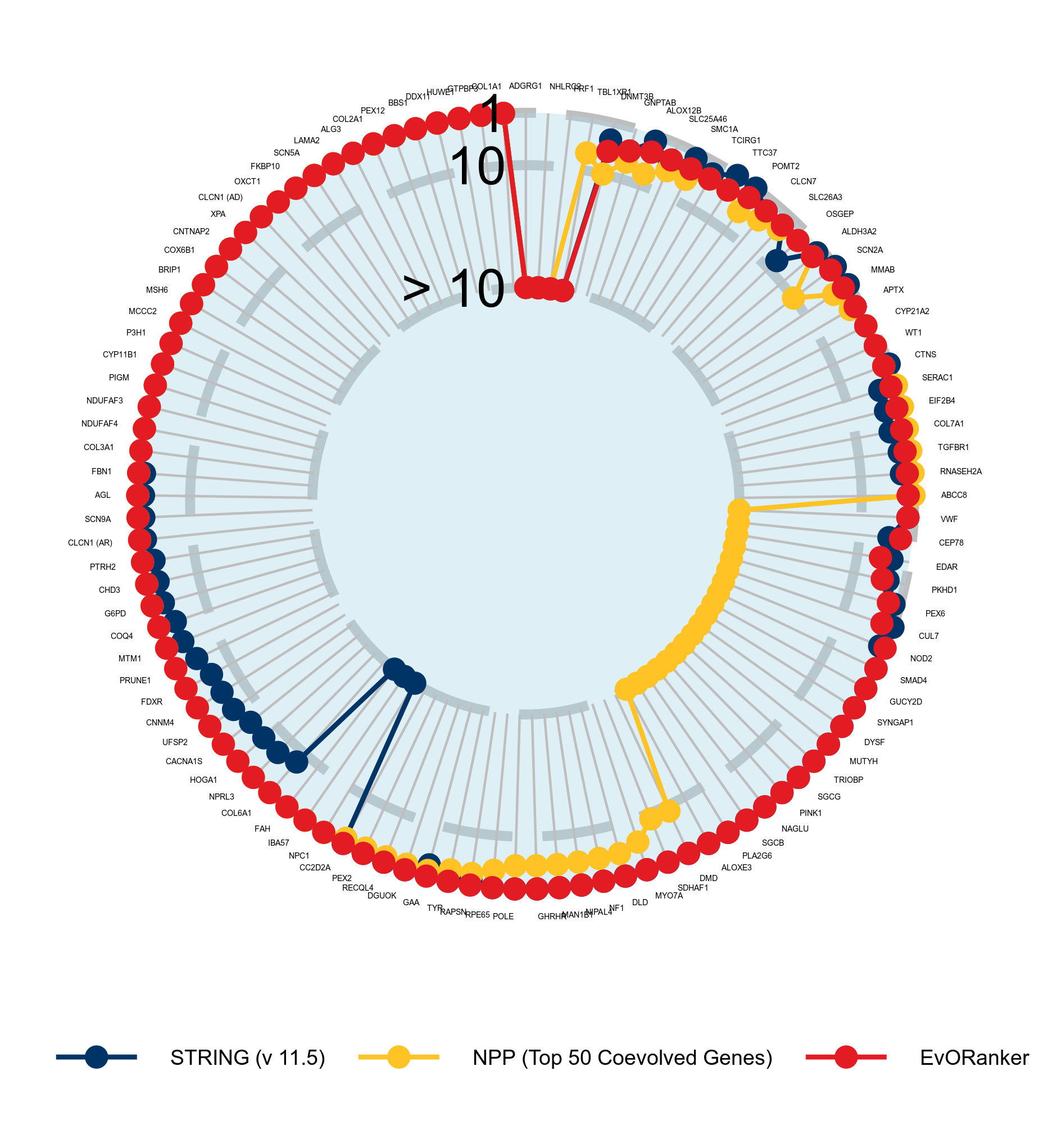


**Figure S6.** Radar plot showing the ranking of the “true” disease-causing gene (top 1, top 10, or NULL) using EvORanker (red), NPP (golden), and STRING (dark blue).


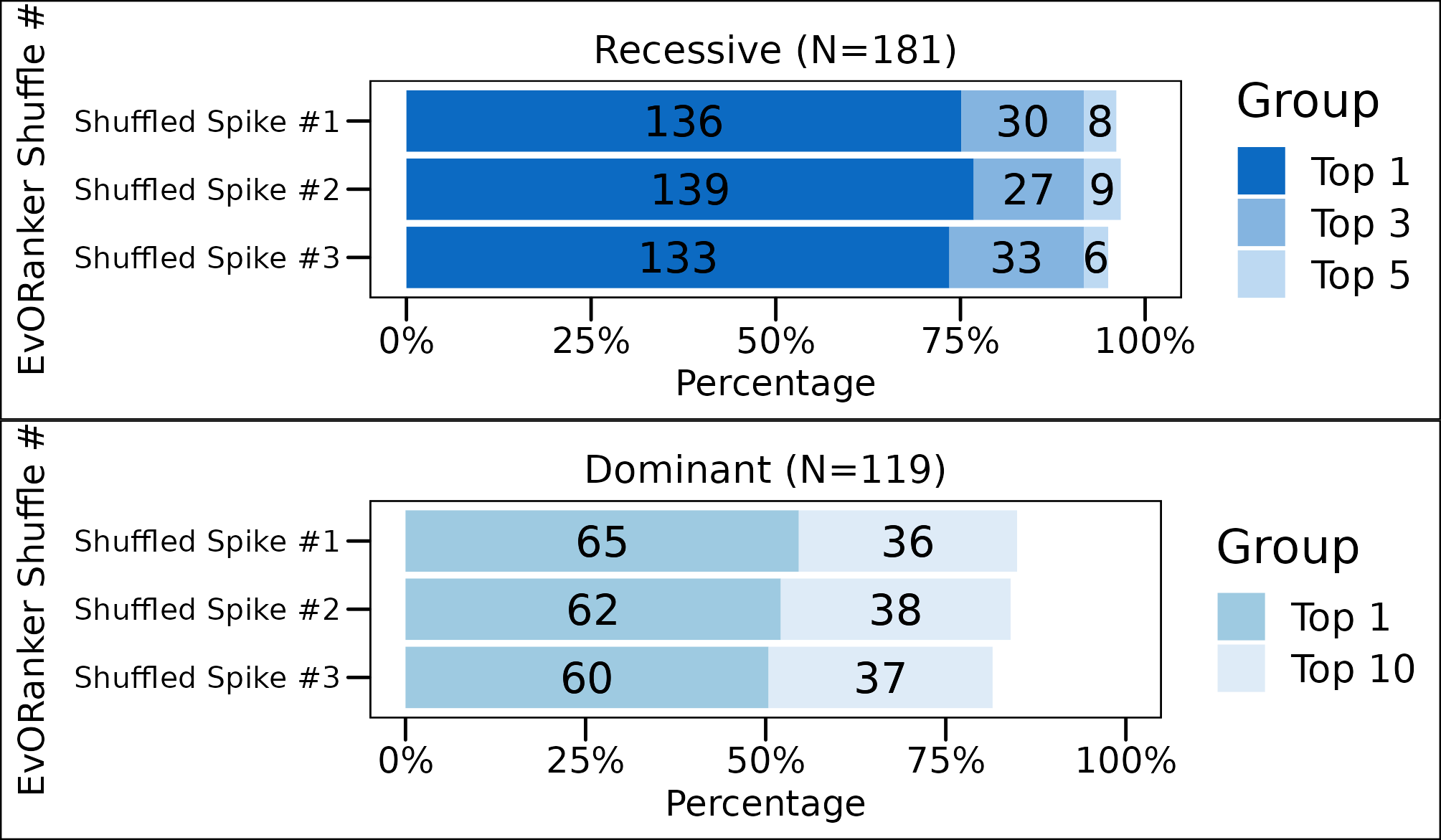


**Figure S7. Evaluating EvORanker Performance across three independent spike shuffles.** The 300 randomly sampled pathogenic/likely pathogenic ClinVar variants were shuffled independently three times across the 300 genomes (Table S2). The goal was to assess the robustness and consistency of EvORanker's performance. Accuracy was gauged by examining the ranking of the 'true' disease-causing gene containing the pathogenic ClinVar variant in relation to other candidate genes. The upper bar plot presents results for autosomal and X-linked recessive cases within the simulated dataset, while the lower bar plot shows results for autosomal and X-linked dominant cases. The y-axis represents the spike shuffle number, and the x-axis displays the percentage of cases where the 'true' disease gene ranked at the top, within the top 3, or within the top 5 genes relative to the other candidate genes for autosomal recessive cases. For dominant cases, the percentage indicates whether the 'true' gene was ranked at the top or within the top 10 genes. The results were consistently similar across all three independent spike shuffles, highlighting the reliability and stability of EvORanker's performance.


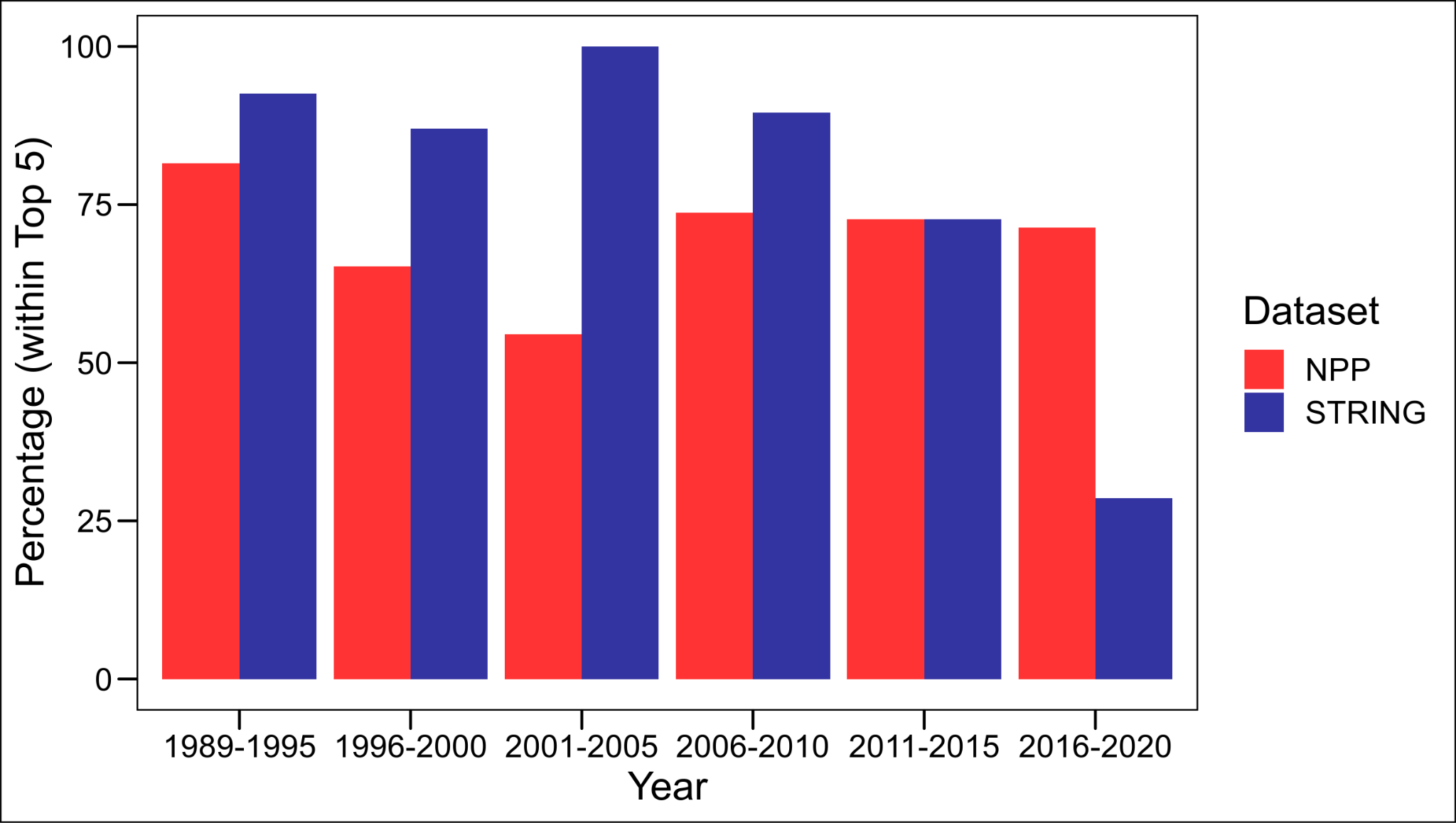


**Figure S8.** **Performance of NPP versus STRING using the 109-patient exome dataset across the years.** The x-axis indicates the years (divided into 5-year windows) in which a gene was described to be associated with a disease phenotype. The y-axis indicates the percentage of the genes that ranked within the top 5 genes relative to the other *patient candidate genes.*


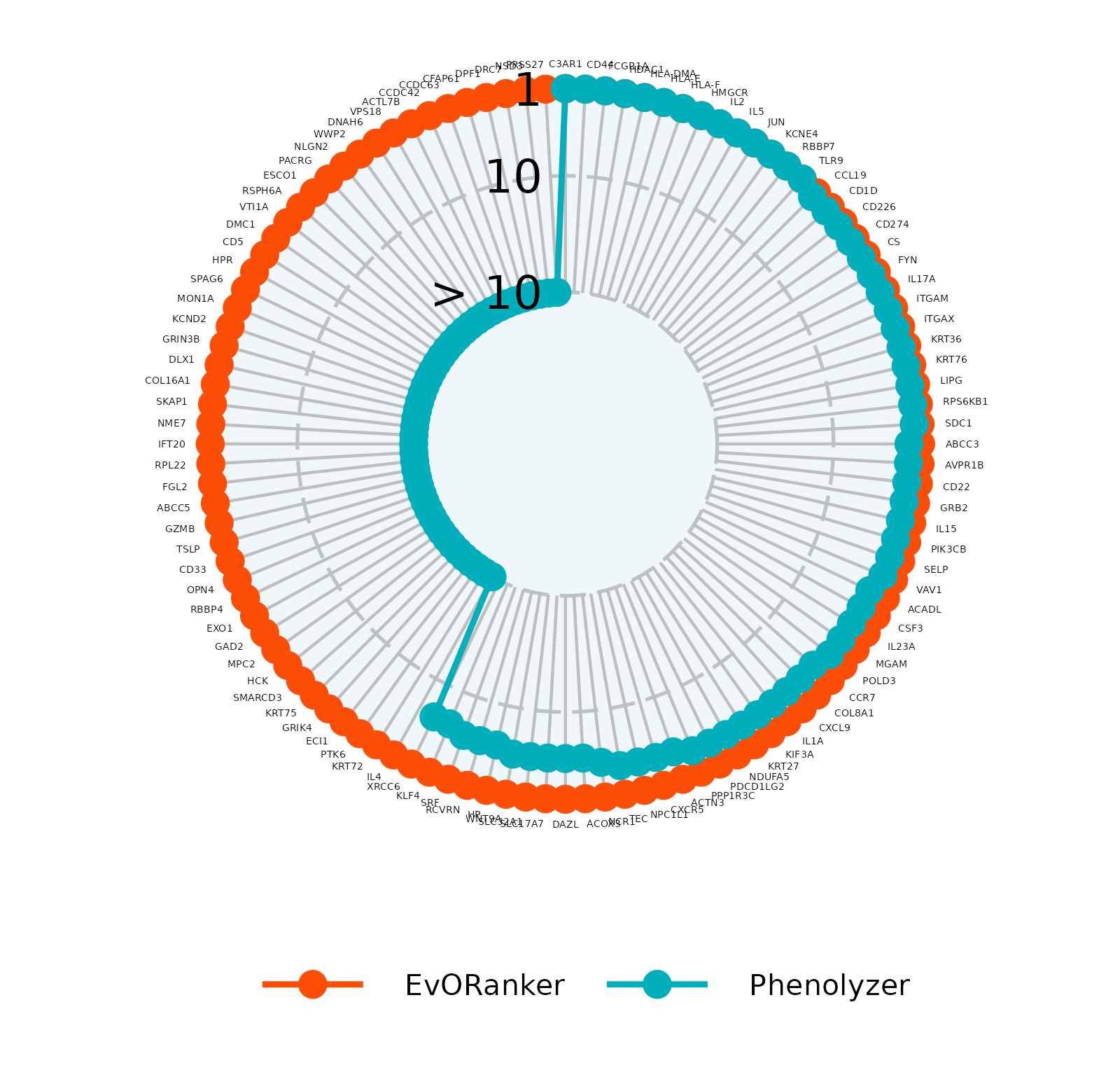


**Figure S9. Comparison of EvORanker and Phenolyzer in identifying true disease gene candidates.** The radar plot shows the cases in which EvORanker ranked the “true” gene at the top according to the phenotypes mapped from the mouse knockout genes. The plot highlights the complementarity of the tools, as EvOranker identified unique sets of candidate genes in addition to the overlapping set.


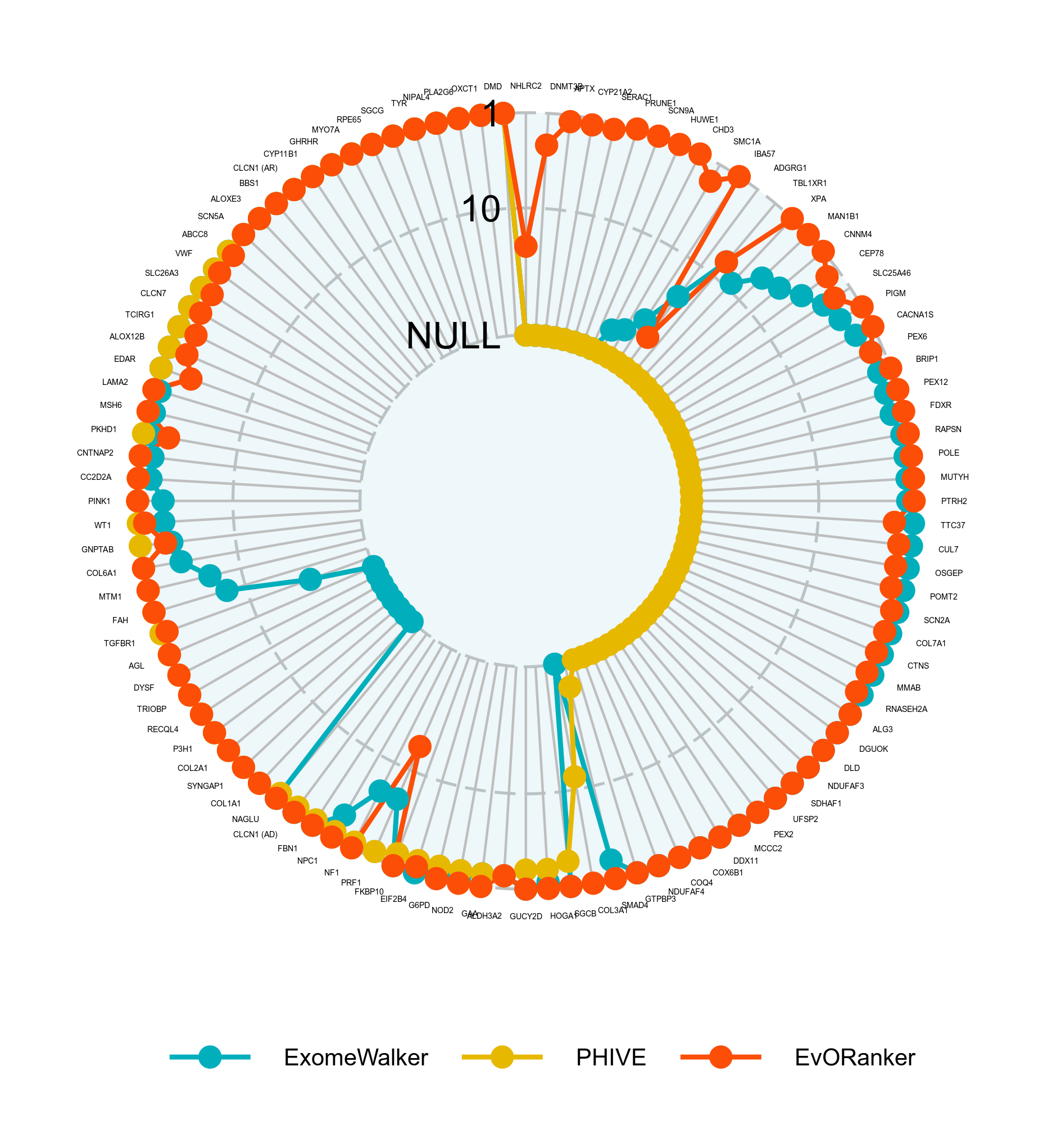


**Figure S10.** Radar plot showing the ranking of the “true” disease-causing gene (top 1, top 10, or NULL) using EvORanker (red), PHIVE (golden), and ExomeWalker (blue).


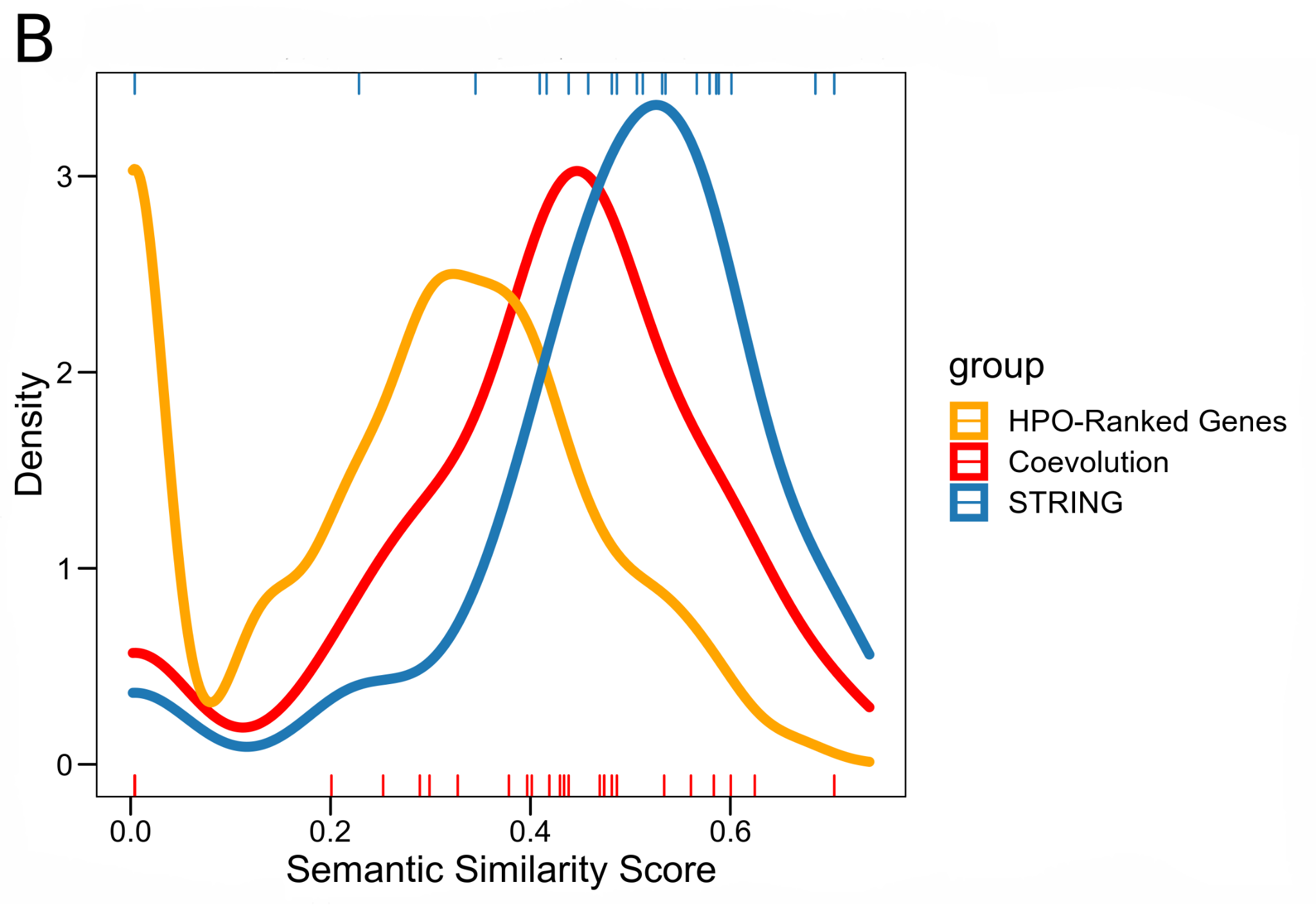


**Figure S11.** **Distributions of the *HPO-ranked genes*, the co-evolved genes, and STRING-interacting genes with *DLGAP2****.* The x-axis indicates the semantic similarity score obtained by the *OntologySimilarity* tool according to the patient’s (II-3, Family 1) phenotypes (HP:0001263, HP:0002357, HP:0000752, HP:0000736). Both coevolved and STRING-interacting genes with *DLGAP2* cluster within genes more related to the patient’s phenotypes.


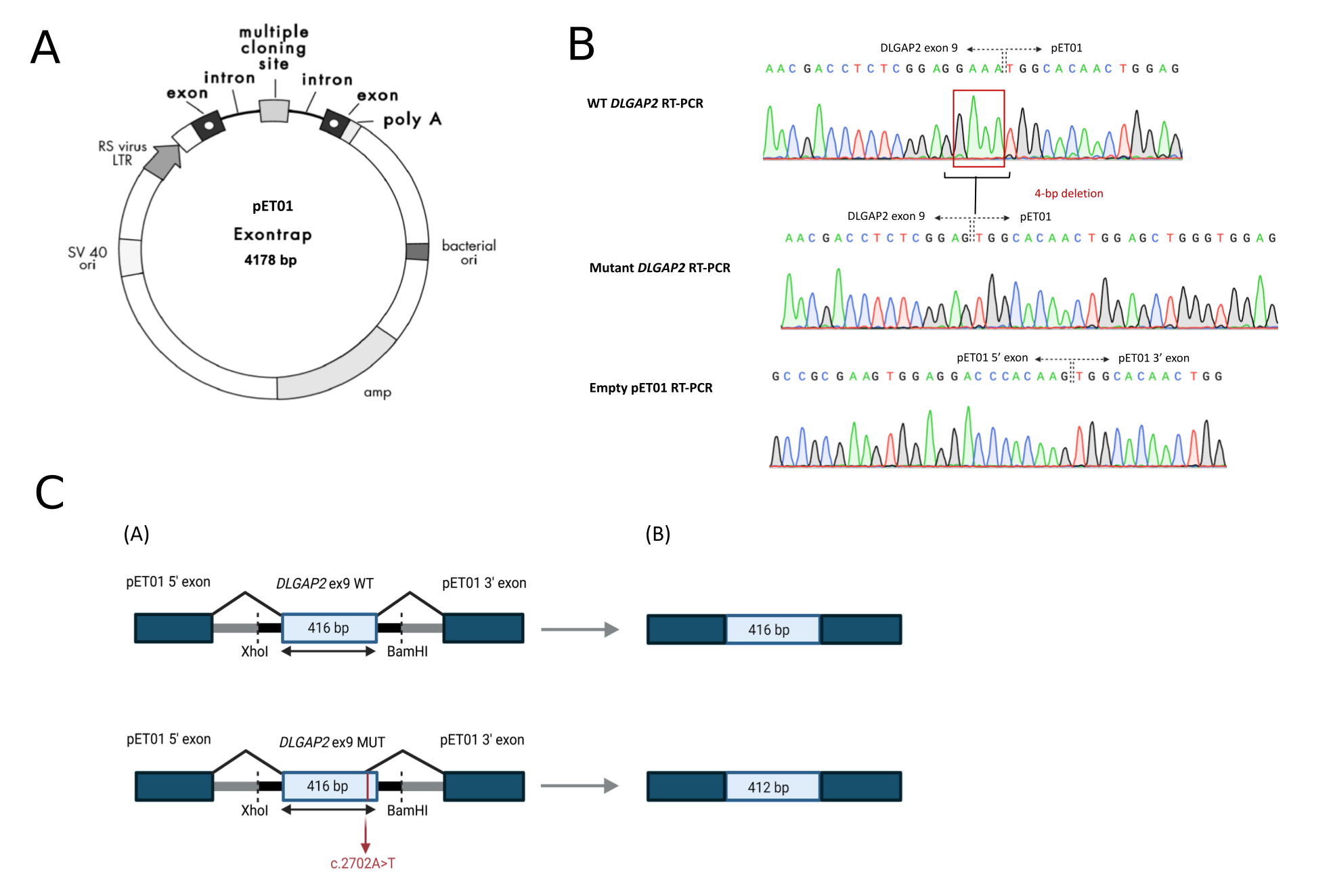


**Figure S12. Effect of *DLGAP2* p.E901V on splicing.** (A) Wildtype and mutant *DLGAP2* exon 9 were inserted into the pET01 Exontrap vector containing 5’ and 3’ exons. (B) ***DLGAP2* p.E901V leads to the activation of a cryptic splice site and aberrant splicing**. Sequencing of the RT-PCR product of the wildtype (top), mutant (middle) construct shows a 4-bp deletion (GAAA del). The empty pET01 vector is shown at the bottom. (C) RT-PCR analysis of spliced transcripts 48-hr post-transfection in HEK293 cells shows normal splicing in the wildtype construct (top panel) and 4-bp deletion in the mutant construct resulting from the activation of a donor cryptic splice site within exon 9 (bottom panel).


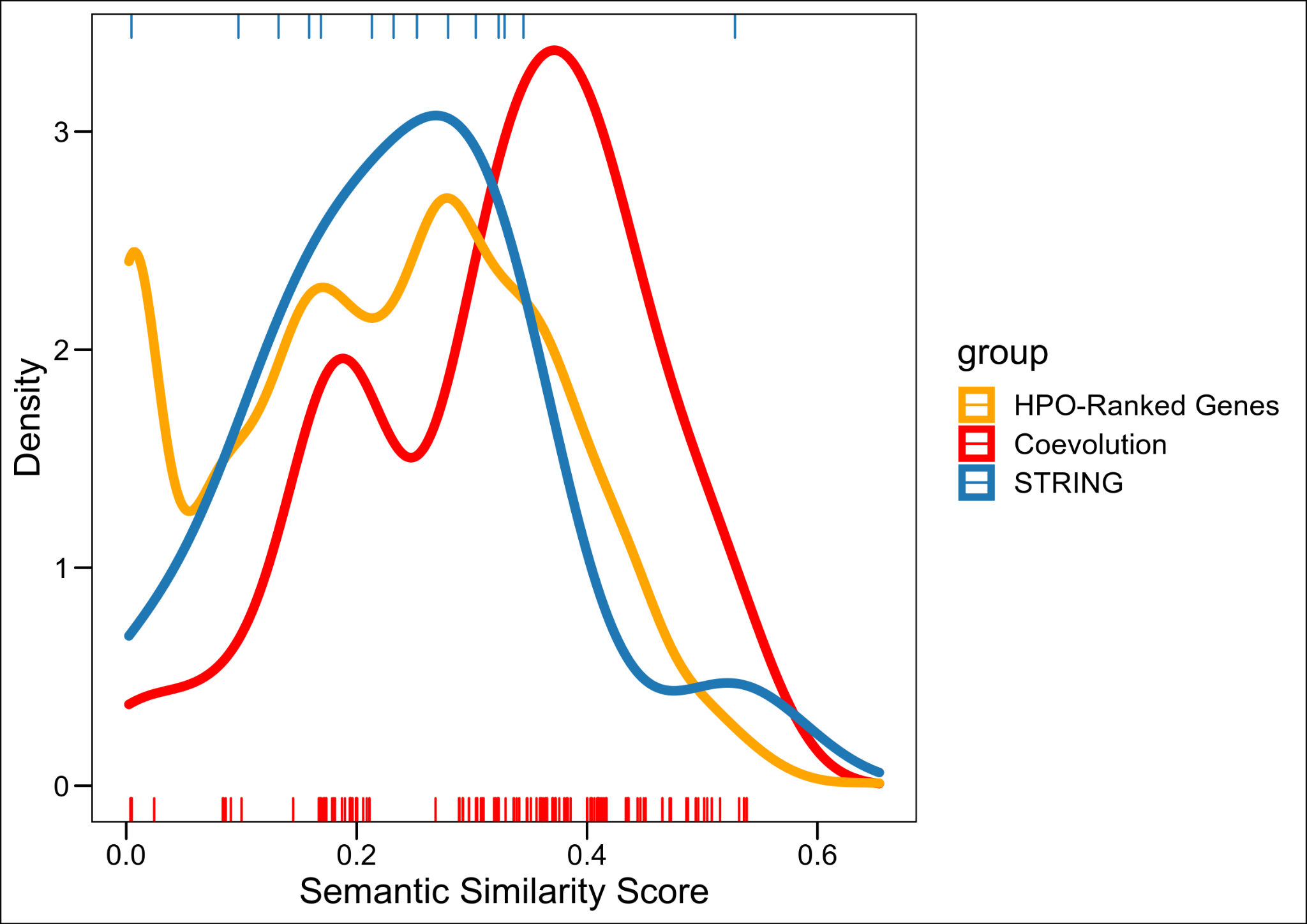


**Figure S13.** **Density distributions of the *HPO-ranked genes*, the co-evolved genes, and STRING-interacting genes with *LPCAT3.*** The x-axis indicates the semantic similarity score according to the patient’s (II-4, Family 2) phenotypes (HP:0001508, HP:0002910, HP:0002574, HP:0002028, HP:0003236, HP:0003202) obtained by the *OntologySimilarity* tool. Only the coevolved genes with *LPCAT3* were clustered within genes more related to the patient’s phenotypes.


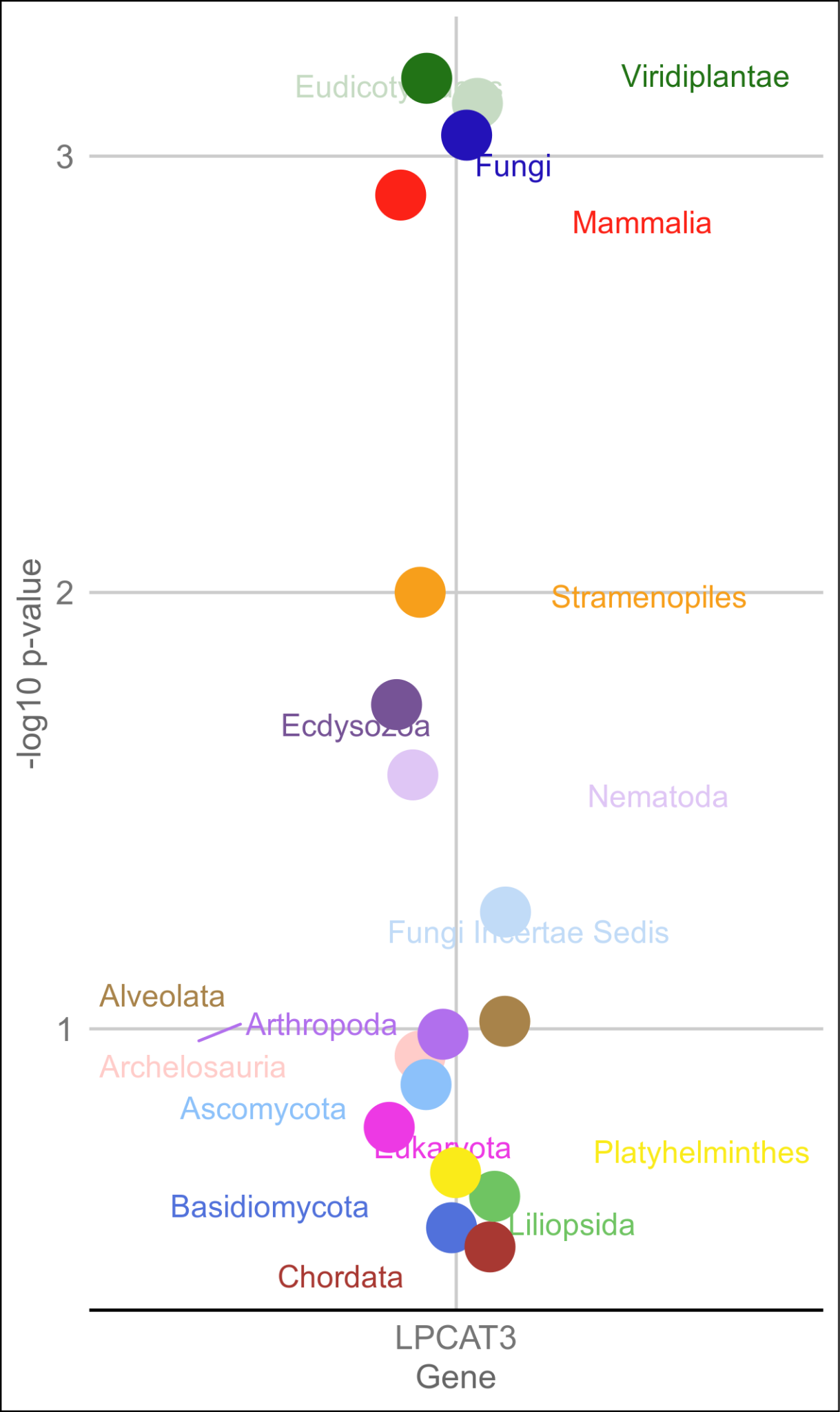


**Figure S14. Clades differentially predict the functional interaction between the *phenotype-related* *genes* and *LPCAT3.*** The X-axis indicates the gene (*LPCAT3*) and the y-axis represents the -log10 of the K-S test p-value in each tested clade. The more significant the p-value, the more enrichment of the *phenotype-related* within the co-evolving genes with *LPCAT3.*


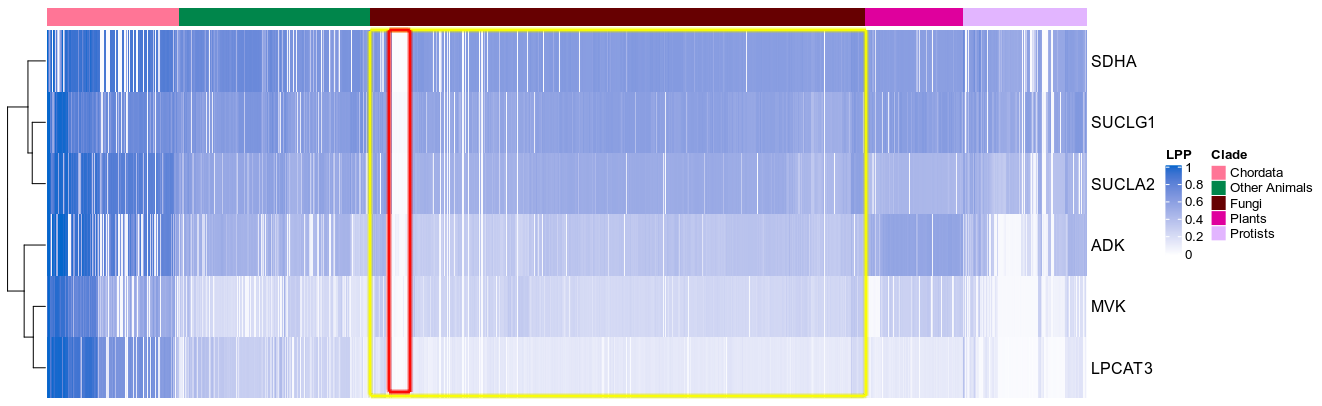


**Figure S15.** **The Phylogenetic profiles of *LPCAT3* and patient HPO-related genes across 1,028 eukaryotes.** Each row represents the normalized BLASTP score of a single gene across 1,028 eukaryotes ordered by their phylogenetic distance from Homo sapiens. Each column represents a species. The rows are hierarchically clustered. The colors in the heatmap indicate the relative degree of conservation between a human protein and its ortholog in certain species (column). Dark blue indicates highly conserved genes, and white indicates poor conservation. The yellow rectangle indicates a strong local coevolution signature detected in the **Fungi** clade. All six genes (*SDHA, SUCLG1, SUCLA2, ADK, MVK, LPCAT3)* were co-lost in the species (27 species) indicated in the red rectangle. *Spraguea lophii, Pseudoloma neurophilia,* and *Ordospora colligata* are examples of the species that lost these three genes.


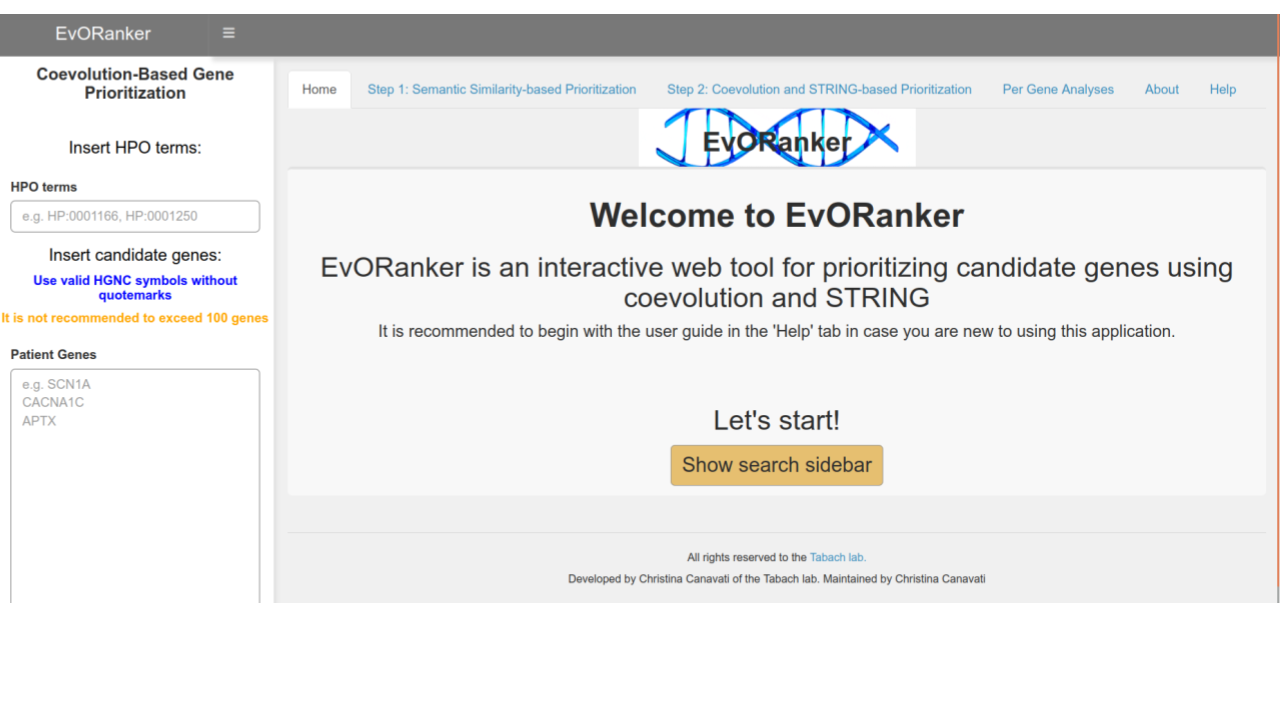


**Figure S16. Homepage of the EvoRanker web interface.** The user needs to input HPO terms describing the patient’s medical condition(s), in addition to a list of patient candidate genes. We recommend that the user selects the most specific term that reflects each patient's phenotypic anomaly. Selecting 2-15 HPO terms covering the affected organ systems is often enough to obtain good results.
